# DeepFGRN: inference of gene regulatory network with regulation type based on directed graph embedding

**DOI:** 10.1093/bib/bbae143

**Published:** 2024-04-04

**Authors:** Zhen Gao, Yansen Su, Junfeng Xia, Rui-Fen Cao, Yun Ding, Chun-Hou Zheng, Pi-Jing Wei

**Affiliations:** The Key Laboratory of Intelligent Computing and Signal Processing of Ministry of Education, School of Computer Science and Technology, Anhui University, 111 Jiulong Road, Hefei, 230601, Anhui, China; The Key Laboratory of Intelligent Computing and Signal Processing of Ministry of Education, School of Artificial Intelligence, Anhui University, 111 Jiulong Road, Hefei, 230601, Anhui, China; Information Materials and Intelligent Sensing Laboratory of Anhui Province, Institute of Physical Science and Information Technology, Anhui University, 111 Jiulong Road, Hefei, 230601, Anhui, China; The Key Laboratory of Intelligent Computing and Signal Processing of Ministry of Education, School of Computer Science and Technology, Anhui University, 111 Jiulong Road, Hefei, 230601, Anhui, China; The Key Laboratory of Intelligent Computing and Signal Processing of Ministry of Education, School of Artificial Intelligence, Anhui University, 111 Jiulong Road, Hefei, 230601, Anhui, China; The Key Laboratory of Intelligent Computing and Signal Processing of Ministry of Education, School of Artificial Intelligence, Anhui University, 111 Jiulong Road, Hefei, 230601, Anhui, China; Information Materials and Intelligent Sensing Laboratory of Anhui Province, Institute of Physical Science and Information Technology, Anhui University, 111 Jiulong Road, Hefei, 230601, Anhui, China

**Keywords:** gene regulatory network, regulatory type, directed graph embedding, correlation analysis

## Abstract

The inference of gene regulatory networks (GRNs) from gene expression profiles has been a key issue in systems biology, prompting many researchers to develop diverse computational methods. However, most of these methods do not reconstruct directed GRNs with regulatory types because of the lack of benchmark datasets or defects in the computational methods. Here, we collect benchmark datasets and propose a deep learning-based model, DeepFGRN, for reconstructing fine gene regulatory networks (FGRNs) with both regulation types and directions. In addition, the GRNs of real species are always large graphs with direction and high sparsity, which impede the advancement of GRN inference. Therefore, DeepFGRN builds a node bidirectional representation module to capture the directed graph embedding representation of the GRN. Specifically, the source and target generators are designed to learn the low-dimensional dense embedding of the source and target neighbors of a gene, respectively. An adversarial learning strategy is applied to iteratively learn the real neighbors of each gene. In addition, because the expression profiles of genes with regulatory associations are correlative, a correlation analysis module is designed. Specifically, this module not only fully extracts gene expression features, but also captures the correlation between regulators and target genes. Experimental results show that DeepFGRN has a competitive capability for both GRN and FGRN inference. Potential biomarkers and therapeutic drugs for breast cancer, liver cancer, lung cancer and coronavirus disease 2019 are identified based on the candidate FGRNs, providing a possible opportunity to advance our knowledge of disease treatments.

## INTRODUCTION

Gene regulatory networks (GRNs) comprise regulators, target genes and directed regulatory associations between regulators and target genes. GRNs can regulate most biological processes [1] and they are one of the reasons for the complexity and variety of life [2]. The reconstruction of GRNs from gene expression profiles is a core issue in systems biology. For example, they can help reveal the essential laws of organism development [3], understand the pathogenesis of complex human diseases [4–6] and identify disease biomarkers and potential drug targets.

An increasing number of computational methods for GRN inference have been developed in recent years, including methods based on information theory [7], ordinary differential equations [8], feature selection [9, 10], prior knowledge [11–13] and deep learning [14]. Information theory-based methods have a limited ability to predict the direction of a GRN. The ordinary differential equation model is more inclined to reconstruct medium-sized GRNs (i.e. GRNs containing several hundred genes) from temporal gene expression data. Feature selection-based methods [1, 3, 10, 15] transform the GRN inference problem into regulator-selecting problem for each gene. However, the prediction accuracy of GRN inference for real species (e.g. *E.coli*) is far from ideal. Prior knowledge-based methods for GRN inference [11, 12] regard ChIP data, biological pathways and protein–protein networks as prior knowledge that can be integrated into algorithms via strong mathematical skills. This type of method can significantly improve prediction accuracy by biasing computational structures toward known biological associations [13]. Deep learning models have been extensively applied to GRN reconstruction from single-cell transcriptomic data [16–20]; however, few methods have been applied to GRN reconstruction from bulk gene expression data [14]. One reason for this is that deep-learning methods require a large number of training samples, which are less available in bulk benchmark datasets. CNNGRN [14] is a convolutional neural network (CNN)-based model for GRN reconstruction using bulk gene expression data. Because of the far-from-adequate labels and fewer time points in gene expression profiles, CNNGRN [14] introduces network structure features into the model and improves the prediction performance of GRN inference, particularly in real species. Interestingly, as in [11, 12], CNNGRN uses known pathways as prior knowledge for GRN inference. [11] integrates prior knowledge of network structure and pathway information into the Lasso method using improved Bayesian information criteria and assigns different prior distributions to gene pairs with or without prior knowledge. [12] designs three methods to encode prior knowledge from the STRING database into a Gaussian graph model for inferring GRN from high-dimensional data, which have the characteristic that the number of genes is larger than the number of biological experiment samples. Moreover, [12] encodes prior knowledge into the target matrix, regularizing terms, and combines the former two methods, respectively. The experimental results show that encoding prior knowledge into both the target matrix and regularizing terms is the most effective. In contrast to [11, 12], CNNGRN uses a prior GRN to obtain gene neighbor information and uses it as a network structural feature to enhance gene pair features. However, CNNGRN does not fully utilize the rich information contained in prior knowledge, such as the directionality of the GRN.

Exploring both the directions and types of regulation between regulators and target genes could help elucidate the pathogenesis of diseases and explore disease treatments. As we all know, regulators play a key role in controlling the expression level of cancer-related genes and are usually regarded as high-value biomarkers and therapeutic targets [21–23]. Currently, various targeted therapies have been established in clinical studies [24], such as inhibiting the DNA binding of regulators [25], which can prevent cancer growth, metastasis and drug resistance to a certain extent [26]. For example, CDK1, HMMR, PTTG1 and TTK may serve as potential biomarkers for the diagnosis, prognosis and targeted therapy [27]. HIF-1$\alpha $ and E2A regulators have the potential for targeted treatment of breast cancer [28, 29]. Overall, these regulators are promising anticancer drug targets. Drugs targeting master regulators have been used to treat human diseases. For instance, sorafenib, regorafenib, lenvatinib and cabozantinib are therapeutic drugs used to treat hepatocellular carcinoma (HCC). They repress the development of HCC through target regulators, such as STAT-1, STAT-3, NFkappaB, Ap1 and HIF-1$\alpha $ [30]. Thus, there is an urgent need to reconstruct fine gene regulatory networks (FGRNs) to lay the foundation for disease treatment. Therefore, in this study, the DeepFGRN model was designed to reconstruct FGRNs with both directions and regulatory types.

The expression profiles of genes with regulatory relationships are usually strongly correlated, which is the basis of traditional GRN inference methods. Although much effort has been made to extract features from bulk gene expression profiles, existing deep learning-based methods have not considered the correlation between regulatory factors and target genes. Hence, we designed a correlation analysis module to extract the correlation embeddings of gene pairs. First, a one-dimensional residual neural network was implemented to fully extract gene expression features, alleviating the gradient vanishing problem caused by low gene expression data dimensions. The correlation embedding between gene pairs can then be obtained via a neural network with gene expression features as inputs, which is conducive to improving the prediction performance of the model.

The topological information of GRNs is vital for GRN inference [14]. One possible reason is that genes performing the same function are generally topologically related. To capture appropriate network structure information, we summarized two challenges that must be addressed: (1) the GRNs of real species are sparse and large. This is because the GRNs of real species usually contain a considerable number of gene nodes, whereas known regulatory associations account for only a small percentage. (2) GRNs are directed graphs. The direction of a graph always contains the primary asymmetric semantic information. To this end, it is necessary for GRN inference to build a directed graph embedding method that aims to obtain low-dimensional dense embedding of the GRN while preserving its asymmetric semantic information. Therefore, the DeepFGRN model builds a generative adversarial network (GAN) with two generators: one for generating source neighbor embeddings and the other for generating target neighbor embeddings. The adversarial learning strategy of the GAN enables the model to learn the almost real neighbor embedding of each gene.

The main contributions of this paper are as follows:

- We propose the DeepFGRN model, which can not only predict directed regulatory associations between regulators and target genes but also identify the regulation type, shedding light on the nature of fine gene regulation mechanisms.
- The correlation analysis module is designed to extract both the gene expression features of regulators and target genes and the correlation embedding between regulatory factors and target genes.
- The bidirectional node representation module is established based on a GAN with two generators. It can not only embed the FGRN into a low-dimensional space, but also capture directed node embedding, including source neighbor and target neighbor embedding.
- We collect nine benchmark datasets and identify potential biomarkers and therapeutic drugs for human diseases using the DeepFGRN model.

## MATERIALS AND METHODS

### Overview of DeepFGRN

**Figure 1:**
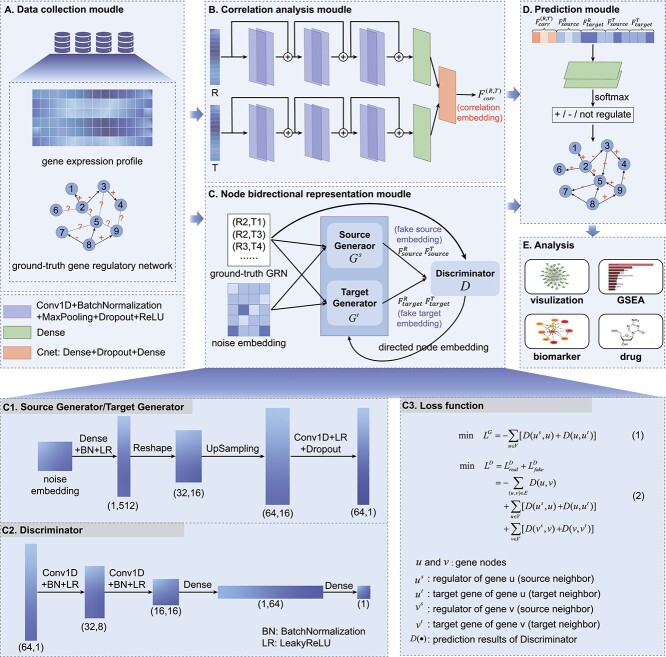
Overview of the DeepFGRN framework. **A**. Data collection. Gene expression data and prior ground-truth GRNs with regulatory types are collected from databases. **B**. Correlation analysis module. The correlation embeddings between regulatory factors and target genes are calculated on the basis of fully extracting features of bulk gene expression data. **C**. Node bidirectional representation module. The source neighbor and target neighbor embeddings of each gene node are generated jointly using GAN with two generators and one discriminator. c1. The architecture of the two generators. c2. The architecture of the discriminator. c3. The loss function of the GAN. **D**. Prediction module. Concatenates the aforementioned features and predicts regulatory association with regulation type between regulators and target genes (activation/inhibition/non-regulation). e. Analysis module. The potential biomarkers and therapeutic drugs associated with a specific disease are discovered via bioinformatics analysis.

DeepFGRN is an end-to-end framework that can infer FGRNs with both the direction and regulation types. DeepFGRN converts the FGRN inference problem into an association-prediction task between regulators and target genes. After extracting the features of the regulator–target gene pairs via deep learning methods, softmax was performed for prediction. Finally, we obtained the regulatory relationships between the regulators and target genes, which could be activated, repressed or non-regulated. The DeepFGRN model consists of five submodules, as shown in Figure 1.

First, since there are no benchmark datasets for FGRN inference, we collected bulk gene expression data and prior regulatory associations with regulation types from databases, as shown in Figure 1a. Then, the gene expression profiles of gene pairs $(R,T)$ were input into the correlation analysis module (Figure 1b) to obtain the correlation embedding vector $F_{corr}^{(R,T)}$, which contains both gene expression features of gene pairs and the correlation information between regulators and target genes.

The incomplete ground-truth GRN was sent to the node bidirectional representation module (Figure 1c), to learn the potential low-dimensional bidirectional representations $F_{source}^{R}$, $F_{target}^{R}$, $F_{source}^{T}$, $F_{target}^{T}$ of each gene pair $(R,T)$. This module comprised two generators and one discriminator [31]. Two generators, $G^{s}$ and $G^{t}$ (Figure 1c1), were used to learn the source and target neighbor embeddings of each gene node, respectively. One discriminator $D$, (Figure 1c2), was established to distinguish whether the generated neighbor node was true or false. Through multiple adversarial games between the generators ($G^{s}$ and $G^{t}$) and discriminator ($D$), the abilities of both the discriminator and generators can be improved until the generators are no longer distinguishable from the real distribution.

We concatenated all of the above features and sent them to the prediction module (Figure 1d). Specifically, the features of a gene pair $(R,T)$ included correlation embedding $F_{corr}^{(R,T)}$ between regulator $R$ and target gene $T$, and the potential source/target neighbor embedding $F_{source}^{R}$, $F_{target}^{R}$, $F_{source}^{T}$, $F_{target}^{T}$ of regulator $R$ and target gene $T$, respectively. The prediction module can predict the effect (activation/inhibition/non-regulation) of the regulator on the target gene. In addition, visualization and bioinformatic analysis (Figure 1e) of the predicted potential FGRNs were performed for the four diseases.

### Data collection

In this section, we collected a benchmark dataset for FGRN inference. Currently, lung, breast and liver cancers are the most common cancers [32]. Coronavirus disease 2019 (COVID-19) can cause respiratory failure [33]. Therefore, there is an urgent need to study the fine gene regulation mechanisms of these human diseases. Additionally, *E.coli* is a common species in the field of GRN inference; hence, we collected benchmark data on the *E.coli* challenge for FGRN inference.

Specifically, we downloaded the gene expression data of *E.coli* and human diseases from the Gene Expression Omnibus (GEO) [34] and Gene Expression Nebulas (GEN) databases [35], respectively. Known gene pairs with both direction and regulatory types were collected from RegulonDB [36], TRRUST V2 [37] and RegNetwork [38]. For each species, we compared the genes in the FGRN with those in the gene expression profiles, and retained the same genes to reconstruct the FGRN. Besides, the DREAM5 challenge [39] network1 was reconstructed using DeepFGRN. Information on the organized benchmark datasets is shown in Table 1, and the data download process is shown in Figure S1.

**Table 1:** Benchmark datasets

Species	Datasets	sourceE	sourceGRN	numG	dimE	numA	numR
DREAM5	network1	DREAM5 challenge	DREAM5 challenge	1643	805	2236	1776
*E.coli*	cold stress	GEO (GSE20305)	RegulonDB	2205	24	2070	2034
*E.coli*	heat stress	GEO (GSE20305)	RegulonDB	2205	24	2070	2034
*E.coli*	lactose stress	GEO (GSE20305)	RegulonDB	2205	12	2070	2034
*E.coli*	oxidative stress	GEO (GSE20305)	RegulonDB	2205	33	2070	2034
Human	COVID-19	GEN (GEND000389)	TRR, Reg	2478	42	6452	1888
Human	Breast cancer	GEN (GEND000024)	TRR, Reg	2478	24	6452	1888
Human	Liver cancer	GEN (GEND000025)	TRR, Reg	2478	10	6452	1888
Human	Lung cancer	GEN (GEND000176)	TRR, Reg	2478	130	6452	1888

Note: sourceE and sourceGRN represent databases that store gene expression data and prior GRN information, respectively, numG is the number of genes, dimE is the dimension of gene expression data, numA and numR represent the number of regulatory associations for known activation types and known repression types, respectively. TRR and Reg are TRRUST V2 and RegNetwork databases, respectively.

Furthermore, $\log _{2}({x+1})$ normalization and min–max normalization were performed on gene expression profiles. The ground-truth networks were preprocessed using one-hot encoding and then regarded as labels for training DeepFGRN.

### Correlation analysis module

The objective of the correlation analysis module is not only to fully extract the gene expression data of the regulator $R$ and target gene $T$, but also to calculate the correlation embedding of the gene pair $(R,T)$.

To learn the appropriate gene expression features from the bulk gene expression profiles, a one-dimensional CNN was used. However, the problem of gradient disappearance was prone to occur when a one-dimensional CNN was used to extract gene expression features. This was because the gene expression data were shorter than the other sequence data. Thus, skip connections were introduced into a one-dimensional CNN. Specifically, the gene expression features of the regulator $R$ and target gene $T$ were extracted using three residual blocks, a global average pooling layer and a dense layer. Each residual block includes a one-dimensional convolutional layer, a batch normalization layer and a MaxPooling layer, and the activation function is ReLU, as shown in Equations 1–4.

The gene expression features of the regulator $R$ and target gene $T$ were then input into Cnet, which was built to calculate the correlation embedding of the gene pair $(R,T)$. Cnet contains two dense layers and a dropout layer conducted to tackle overfitting, as shown in Equation 5. 


(1) \begin{align*} & A=MP(BN(RELU(y_{i-1}^{R}*w_{i}^{R1}+b^{R1}))),\ i=1,2,3 \end{align*}

(2) \begin{align*} B=MP(BN(RELU(A*w_i^{R2}+b^{R2}))),\ i=1,2,3 \end{align*}

(3) \begin{align*} & shortcut=MP(BN(RELU(y_{i-1}^{R}*w_{i}^{\mathfrak{s}}+b^{\mathfrak{s}}))),\ i=1,2,3 \end{align*}

(4) \begin{align*} & y_{i}^{R}=shortcut+B,\ i=1,2,3 \end{align*}

(5) \begin{align*} & Corr=Dense(Dropout(Dense(Flatten(concat(y_{3}^{R},y_{3}^{T}))))) \end{align*}

where $\ast $ is convolution operation. $y_{i-1}^{R}$ is gene expression profile of regulator $R$, which is also the input to the first convolution layer in the $i$-th residual block. $w_{i}^{R1}$ and $b^{R1}$ are weights matrix and bias vector of the first convolution layer in the $i$-th residual block, respectively. $A$ is the output of the first convolution block in the $i$-th residual block, and the input of the second convolution layer in the $i$-th residual block. $w_{i}^{R2}$ and $b^{R2}$ are weights matrix and bias vector of the second convolution layer in the $i$-th residual block, respectively. $B$ is the output of the second convolution block in the $i$-th residual block. $shortcut$, $w_{i}^{s}$ and $b^{s}$ are weights matrix and bias vector of the skip connection in the $i$-th residual block, respectively. Finally, we can get the embedding $y_{3}^{R}$ of the regulator. Similarly, we input the gene expression profile of the target gene into the above process, then the embedding $y_{3}^{T}$ of the target gene is obtained. $Corr$ is the correlation embedding of a gene pair $(R,T)$.

### Node bidirectional representation module

#### Network architecture

The network architectures of the source ($G^{s}$) and target ($G^{t}$) generators are the same. The dimension of directed graph embedding generated via $G^{s}$ or $G^{t}$ is a hyper-parameter that can be set to different dimensions depending on the dataset. To acquire the optimal dimension of directed graph embedding, we conducted a feature dimension optimization experiment (see Figure S3 and Table S4). Here, the network architectures of generator $G^{s}$/$G^{t}$ and discriminator $D$ were introduced using 64-dimensional directed graph embedding as an example, as shown in Figure 1c1 and Figure 1c2.

For generators $G^{s}$/$G^{t}$, the noise embedding was initialized through Gaussian distribution. Then, the noise embedding was transformed into a tensor with the shape of (1,512) by a dense layer and batch normalization layer, and the activation function is LeakyReLU. After the shape of the above tensor was changed to (32,16), the one-dimensional upsampling layer was implemented to transform the tensor with the shape of (32,16) into (64,16). Finally, a one-dimensional convolution block was applied to obtain the fake neighbor embedding (64,1). The input received by the discriminator was either source neighbor embedding, target neighbor embedding generated by the generator, or embedding of a real gene pair. The input was passed through two one-dimensional convolution blocks and two dense layers, and sigmoid was used to predict the probability that the sample was real. The above one-dimensional convolution blocks are the one-dimensional convolution layer and batch normalization layer, and the activation function is LeakyReLU.

#### Loss function

Given a directed graph $DG=\{V,E\}$, $V$ is the set of nodes and $E$ is the set of directed edges. For nodes $u,v\in V$, $(u,v)\in E$ represents the directed edge from $u$ to $v$. $u^{s}$ and $u^{t}$ represent the false source neighbor $u^{s}$ and false target neighbor $u^{t}$ of gene $u$, respectively, and $D(\bullet )$ represents the probability of the discriminator predicting a sample.

The loss functions of this module were designed based on the Wasserstein distance [40], as shown in Equations 6 and 7. The goal of the generators is to generate source neighbors and target neighbors of gene nodes, which are real enough to deceive the discriminator. In other words, the discriminator is expected to predict the probability that $u^{s}$ and $u^{t}$ are real samples as high as possible, so the terms $a$ and $b$ of Equation 6 are as high as possible. Therefore, the optimization of the generator can be completed by minimizing Equation 6. The goal of the discriminator is to distinguish the real sample from the fake sample. In other words, the discriminator wants its prediction probability of the real sample $(u,v)$ to be as high as possible, and its prediction probability of the samples generated by the generator $(u^{s},u)$, $(u, u^{t})$, $(v^{s},v)$, $(v, v^{t})$ to be as low as possible. Then, item $a$ of Equation 7 is as high as possible, and items $b-e$ are as low as possible. Therefore, the discriminator can be optimized by minimizing Equation 7. 


(6) \begin{align*} &\min L^{G}=-\sum_{u\in V}[\underbrace{D(u^{s},u)}_{a},\underbrace{D(v^{t},v)}_{b}] \end{align*}

(7) \begin{align*} &\begin{aligned} \min L^{D}&=L_{real}^{D}+L_{fake}^{D}\\&=-\sum_{(u,v)\in E}\underbrace{D(u,v)}_{a} \\&+\sum_{v\in V}[\underbrace{D(u^{s},u)}_{b}+\underbrace{D(u,u^{t})}_{c}] \\&+\sum_{v\in V}[\underbrace{D(v^{s},v)}_{d}+\underbrace{D(v,v^{t})}_{e}] \end{aligned} \end{align*}

### Prediction module

Each sample contains features and label of a gene pair $(R,T)$. Features include correlation embedding $F_{corr}^{(R,T)}$ of the regulator $R$ and target gene $T$, the potential source/target neighbor embedding $F_{source}^{R}$, $F_{target}^{R}$, $F_{source}^{T}$, $F_{target}^{T}$ of the regulator $R$ and target gene $T$, as shown in Equation 8. Then, the aforementioned features and one-hot encoded labels are input into the prediction module (Figure 1d), which includes a one-dimensional convolution layer, a batch normalization layer, a MaxPooling layer and two dense layers; the last dense layer is classified by Softmax, as shown in Equations 9–11. Finally, it can be predicted that the effect of the regulator on the target gene is to activate/repress/not regulate. In addition, this module is trained via cross-entropy loss (Equation 12) which updates based on the Adam optimizer. 


(8) \begin{align*} & allFe=concat(F_{corr}^{(R,T)},F_{source}^{R},F_{target}^{R},F_{source}^{T},F_{target}^{T}) \end{align*}

(9) \begin{align*} &O_{1}={BN}(\mathit{RELU}(\mathit{allFe*}W_{p}+b_{p})) \end{align*}

(10) \begin{align*} &O_{2}=MP(O_{1}) \end{align*}

(11) \begin{align*} &O_{3}=Dense(Dense(O_{2})) \end{align*}

(12) \begin{align*} &Loss=-\frac1n\sum_{i=1}^{n}\sum_{c=1}^{m}[Y_{ic}^{true}log(Y_{ic}^{pred})] \end{align*}

where $allFe$ is a feature of a gene pair $(R,T)$, $W_{p}$ represents the weight matrix of the convolutional layer, $b_{p}$ is the bias vector of the prediction module and $O_{1}$, $O_{2}$ and $O_{3}$ are the outputs of the convolutional, MaxPooling and prediction layers, respectively. $n$ and $m$ are the number of training samples and classes, respectively. $Y_{ic}^{true}$ and $Y_{ic}^{pred}$ represent the true labels and prediction probabilities of a gene pair, respectively.

## RESULTS AND DISCUSSION

### Experimental settings

To evaluate the prediction performance of the DeepFGRN model, we implemented 5-fold cross-validation (FCV) methods 10 times, and calculated the average indexes, such as the area under the receiver operating characteristic curve (AUC), Matthews correlation coefficient (MCC), F1-score (F1), recall and precision, which were positively correlated with the prediction performance of the model, where the AUC was the most important index (see Text. S1). Because of the slight class imbalance in the dataset, we utilized the $class\_weight$ in sklearn to assign weights to different types of samples [41], thereby enhancing the role of a class with a small sample size and reducing the role of a class with a large sample size. Early stop mechanisms were used to reduce overfitting. Furthermore, we designed several experiments to select the optimal network architecture for the node bidirectional representation module (see Tables S2 and S3), correlation analysis module (see Table S1) and optimal feature dimensions (see Figure S3 and Table S4).

### DeepFGRN achieves superior performances in both FGRN inference and GRN inference

To validate the effectiveness of DeepFGRN in reconstructing GRNs from bulk gene expression profiles, we compared DeepFGRN with existing methods in both regular GRN and FGRN reconstructions.

**Figure 2:**
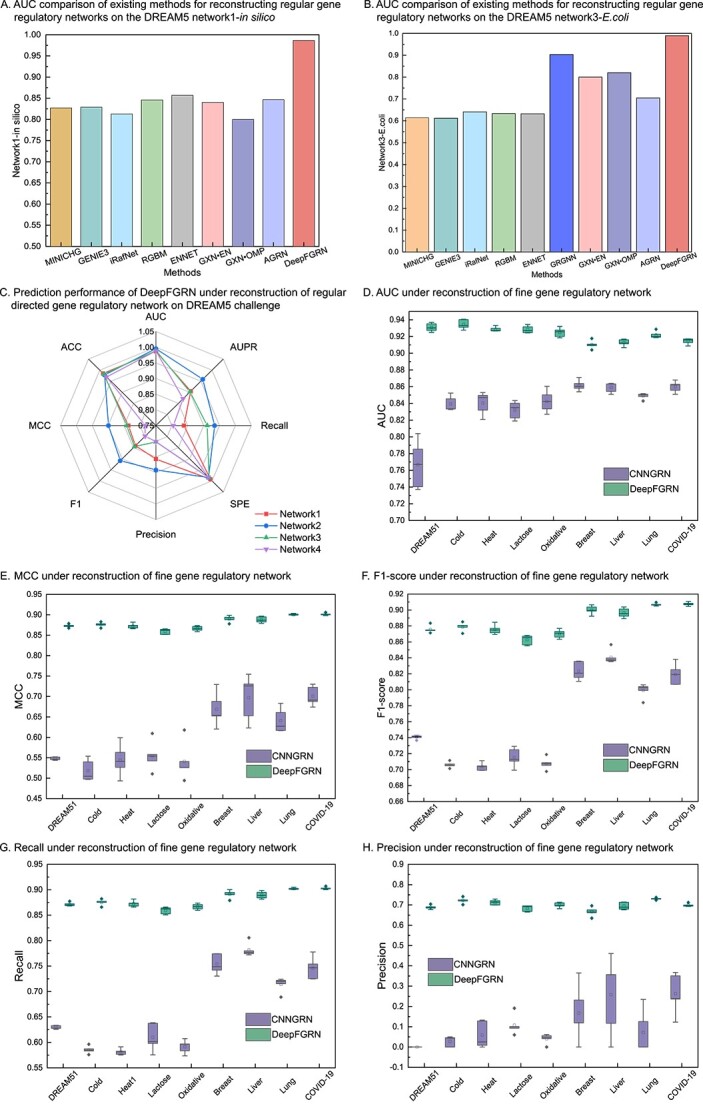
Comparison results of the DeepFGRN model with existing state-of-the-art methods. **A**–**B**. Results of DeepFGRN compared with state-of-the-art methods for regular GRN reconstruction from bulk gene expression profiles. **C**. Prediction performance of DeepFGRN for regular GRN reconstruction on more datasets. **D**–**H**. Results of DeepFGRN compared with CNNGRN for FGRN reconstruction on nine datasets.

To validate the performance of DeepFGRN in regular GRN (without regulation type) inference, we selected state-of-the-art methods for regular GRN reconstruction from bulk gene expression data, including MINICHG [42], GENIE3 [10], iRafNet [43], RGBM [44], ENNET [45], GRGNN [46], GXN$\bullet $EN [47], GXN$\bullet $OMP [47] and AGRN [48]. For comparison purposes, we modified the last dense layer in the prediction module of the DeepFGRN model. Specifically, the number of neurons was changed from three to two, and the other modules were unchanged so that binary classification could be completed. Experimental results on the baseline dataset of the DREAM5 challenge (Table S5) are shown in Figure 2a-b and Table S6. The experimental results showed that the AUC of DeepFGRN were significantly higher than those of the other methods, followed by GRGNN [46], and the results of the other methods were not ideal. This is because most of these methods use feature selection to reconstruct GRNs, and one of their characteristics is to search for candidate regulators for each gene separately. However, a GRN is a result of the cooperation of many genes rather than a single gene. Thus, this type of local method is less suitable for GRN inference than global methods (e.g. GRGNN [46] and DeepFGRN). Although the GRGNN [46] uses a graph neural network to capture the network structure features of the GRN, it does not consider the direction of the GRN, which is another significant characteristic of the GRN. Therefore, DeepFGRN is superior to GRGNN [46]. Overall, compared with existing methods, DeepFGRN has certain advantages in predicting regular GRN.

In addition, we applied DeepFGRN to additional datasets to infer regular GRNs, including four datasets in the DREAM5 challenge. The results are presented in Figure 2c and Table S7. It can be found that the differences in AUC, SPE and ACC across the four networks are slight, and these values are very high. In contrast, the differences in F1-score, Recall and MCC were relatively large. Across all metrics, the performance on Network 2 was the best, that on Network 4 was the worst and those on Networks 1 and 3 were approximately the same. Because the AUC value is the index that best reflects the prediction performance of the models in binary classification tasks, we can assume that DeepFGRN can effectively predict all four networks. The four networks in the DREAM5 challenge exhibited different characteristics (see Table S5). The dimensions of the gene expression data of network1 and network3 were higher (805 dimensions). The gene expression data of network2 had low dimensions (160 dimensions) and few known associations. Network4 contained the largest number of genes. DeepFGRN had high AUC values across all four networks, indicating that it is robust and suitable for multiple types of GRNs. There are two reasons for this finding. DeepFGRN built a node bidirectional low-dimensional representation module for embedding sparse and large GRNs into a low-dimensional dense space, and designed a correlation analysis module for extracting expression patterns from gene expression data of varying lengths.

To validate the performance of DeepFGRN in FGRN inference (with both direction and regulation types), we conducted the following experiments. To the best of our knowledge, very few deep-learning-based studies have been devoted to predicting FGRNs from bulk gene expression profiles. Feature selection-based methods may not be able to predict FGRNs. Therefore, we improved the CNNGRN model [14], which is a deep learning-based method for reconstructing regular GRNs (without regulation type). Specifically, we modified only the output of the CNNGRN, leaving other model details unchanged, for a fair comparison of the FGRN reconstruction. The nine datasets were then fed into the CNNGRN model for FGRN prediction. Figure 2d–h show the results of 10 times FCVs. The results indicated that DeepFGRN significantly outperformed CNNGRN in terms of prediction performance. In addition, the standard deviation of DeepFGRN was smaller than that of CNNGRN, indicating that DeepFGRN was more robust. The possible reasons for this are as follows: (1) DeepFGRN addresses the gradient disappearance problem by introducing a skip connection, which CNNGRN ignores. (2) DeepFGRN considers the correlation between regulators and target genes, which is important for GRN reconstruction. (3) With respect to network structure features, DeepFGRN adds the inherent characteristics of GRNs, namely, directivity, large size and sparsity.

In conclusion, the results of all the experiments confirmed that DeepFGRN can not only infer FGRNs effectively, but also infer regular GRNs effectively.

### The effect of correlation analysis module

**Figure 3:**
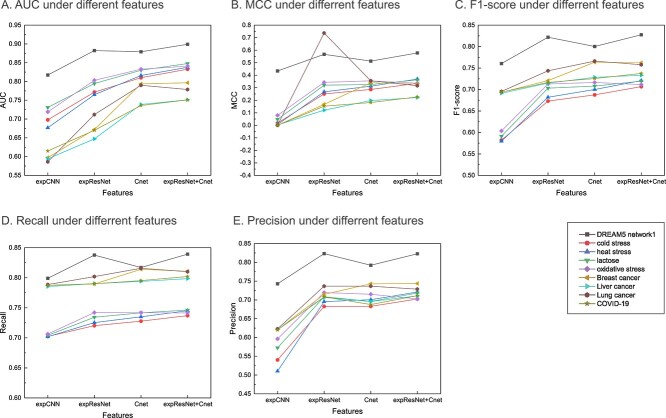
The effect of correlation analysis module on FGRN inference. **A**–**E**. Average results of 10 times FCV for FGRN inference using different gene expression features.

To confirm that calculating the correlation between regulator and target genes promoted FGRN inference, four models were used. (1) expCNN: Only a one-dimensional CNN was used to extract the features of the gene expression data. (2) expResNet: Only one-dimensional ResNet (three residual blocks and one fully connected layer) is used to extract the features of gene expression data. (3) Cnet: Only Cnet was utilized to extract the correlation embedding between the regulators and target genes. (4) expResNet+Cnet: A one-dimensional ResNet and Cnet are jointly used. It should be noted that the aforementioned experiments did not use a node bidirectional representation, and a flowchart of the above experiments is shown in Figure S2. As shown in Figure 3, the results show that expResNet outperformed expCNN on all nine datasets, indicating that one-dimensional ResNet can solve the problem of gradient vanishing that CNNs cannot solve. Furthermore, the results of Cnet were higher than those of expResNet, suggesting that calculating the correlation between regulators and target genes can improve the prediction accuracy of FGRN inference. This is because the expression profiles of two genes that have a regulatory relationship are usually strongly related. Therefore, considering the correlation of gene pairs can improve the prediction performance. In addition, because the difference between the experimental results of expResNet+Cnet and Cnet was not obvious, we combined the correlation analysis module with the node bidirectional representation module in the next section to select the optimal correlation analysis module.

### The effect of node bidirectional representation module

The node bidirectional representation module can embed a large and sparse FGRN into a low-dimensional space and preserve the directed network structure information simultaneously, which can facilitate the prediction performance of the model. To verify this, we conducted experiments.

**Figure 4:**
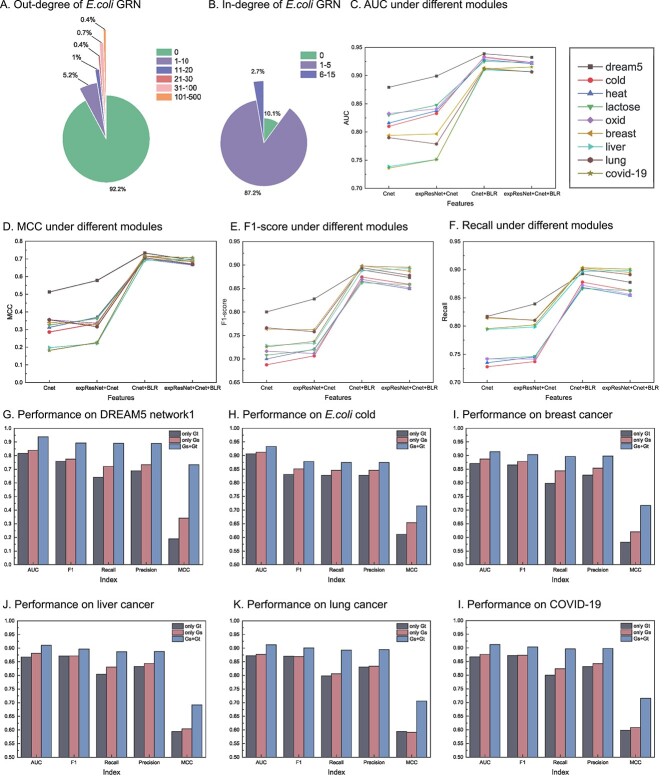
Analysis of node bidirectional representation module. A-B. The out-degree and in-degree of prior FGRN of *E.coli*. **C**–**F**. Average results of ten times FCV for FGRN inference using four features. **G**–**L**. Average results of 10 times FCV for FGRN inference.

The observed FGRNs of *E.coli* and other species have high sparsity; that is, there are a large number of nodes whose out-degrees or in-degrees are low, or even zero, as shown in Figure 4a and 4b. It is apparent that the in-degrees and out-degrees of almost all nodes are low, of which nodes of zero out-degree account for 92.2$\%$ and nodes of in-degree below 5 account for 97.3$\%$. Thus, FGRN of *E.coli* was large and sparse. Utilizing a sparse and large graph as network topology information directly increases the computational complexity and noise. Therefore, a node bidirectional representation module was introduced to cope with the sparsity, largeness and directivity of FGRNs.

To demonstrate the effectiveness of the node bidirectional representation module for FGRN inference, we conducted 10 times FCV based on different features. There were four different feature combinations: (1) Cnet: correlation embedding only. (2) expResNet+Cnet: Both gene expression features were learned via one-dimensional ResNet and correlation embedding. (3) Cnet+BLR: Both correlation embedding and node bidirectional representation were learned using the node bidirectional representation module. (4) expResNet+Cnet+BLR: a combination of gene expression features, correlation embedding, and bidirectional node representation. The experimental results are shown in Figure 4c–f (all results are shown in Figure S4). The results of Cnet+BLR and expResNet+Cnet+BLR were significantly higher than those of Cnet and expResNet+Cnet, indicating that the introduction of a node bidirectional low-dimensional representation is essential for improving the model prediction performance. Because GRN is a type of directed graph, this module uses source and target generators to learn the information of the source and target neighbors of each gene, respectively. Owing to the high sparsity of the GRN, this module uses adversarial strategies to iteratively learn the true distribution of each gene, even for nodes without any known neighbors. Finally, regardless of whether the GRN is a large graph with thousands of genes or a small graph with only a few hundred genes, this module can set the directed network embedding of each gene in the GRN to a low-dimensional vector, such that the GRN can be embedded in the low-dimensional space while capturing the structural information of the GRN network, reducing the computational complexity. In addition, expResNet+Cnet+BLR exhibited slightly lower experimental results than Cnet+BLR, possibly because of the noise that can occur when there are too many features, which slightly reduces the predictive performance of the model. Therefore, the DeepFGRN model combines the correlation embedding representation and node bidirectional low-dimensional representation as the final features of the regulator and target genes.

To further demonstrate the influence of directivity on FGRN inference, we performed experiments on nine datasets in which only the bidirectional representation of nodes differed: (1) only Gs: Only source neighbor representation, (2) only Gt: Only target neighbor representation and (3) Gs+Gt: Both source neighbor representation and target neighbor representation. The results are shown in Figure 4g–l (all the results are shown in Figure S4). It is evident that the experimental results of Gs+Gt are the best, which demonstrates that the prediction performance can be significantly improved by considering the inherent directivity of the FGRN when extracting network structural features. A GRN is a directed graph, which means that each gene node has both source and target neighbors, and ignoring any neighbor is not feasible.

### Potential biomarkers and drugs analysis

To demonstrate the significance of FGRN inference in human disease treatment, we identified potential biomarkers and drugs for breast cancer, liver cancer, lung cancer and COVID-19.

**Figure 5:**
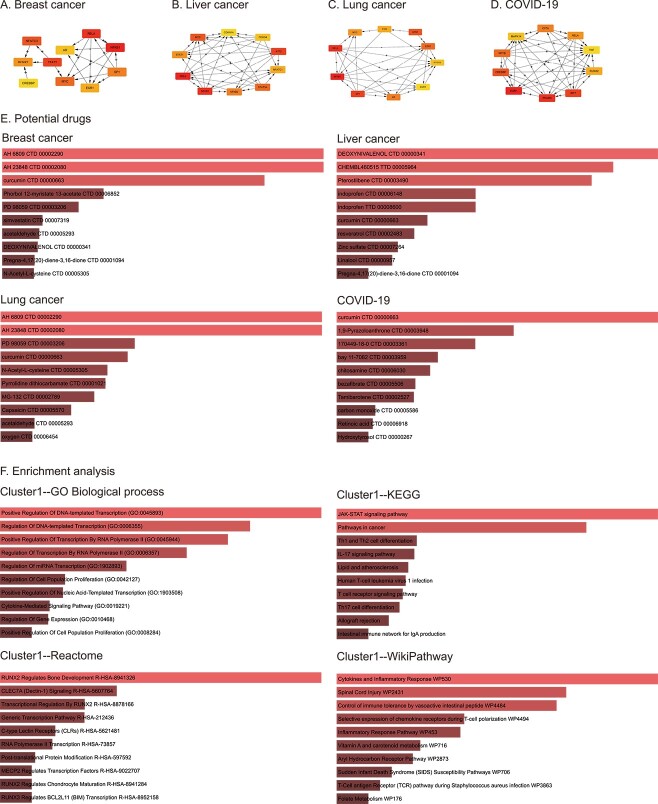
Potential biomarkers and drugs analysis. **A**–**D**. Hub genes of four diseases based on MCC. **E**. The top 10 potential drugs for breast cancer, liver cancer, lung cancer and COVID-19 based on DsigDB. **F**. Enrichment analysis of the first functional module of breast cancer.

We used Cytoscape [49] to visualize the candidate FGRN (see Text S2) and then calculated the MCC scores for the four diseases using Cytohubba [50]. The top 10 hub genes were obtained according to the MCC scores. As a result, the potential biomarkers for four diseases are obtained, as shown in Figure 5a–d. Furthermore, the Enrichr [51–53] and DSigDB databases [54] were combined to identify candidate therapeutic drugs for the four diseases based on the hub genes. The top 10 candidate drug compounds according to *P*-values are shown in Figure 5e, and all the results are shown in Table S8. Taking breast cancer as an example, the top 10 drugs were AH 6809 [55], AH 23848 [55], curcumin [56], phorbol 12-myristate 13-acetate [57], PD 98059 [58], simvastatin [59, 60], acetaldehyde [61], deoxynivalenol, pregna- 4,17(20)-diene-3,16-dione and N-acetyl-L-cysteine. Literature shows that the first seven compounds have been shown to play an important role in breast cancer treatment, and the remaining drug compounds are worthy of further exploration.

Besides, DeepFGRN can be used to reconstruct biologically meaningful FRGNs from gene expression profiles. Specifically, we performed an enrichment analysis of the functional modules obtained via MCODE [62], and the results are shown in Table S9. Because the genes in FGRNs with higher node centrality are more likely to participate in key biological processes [17], we selected important functional modules for enrichment analysis. Enrichment analysis based on the GO, KEGG, Reactome and WikiPathway databases was conducted. The enrichment analysis results of the first functional module of breast cancer are shown in Figure 5f. The genes in this functional module participate in many vital biological processes, including various transcriptional regulation (GO:0045893, 0006355, 0045944, 0006357 and 1902893). KEGG enrichment analysis revealed that this gene set is involved in cancer pathways and deserves further exploration. In addition, Reactome and WikiPathway analyses showed that these genes were related to the regulation of bone development, bone marrow injury and the inflammatory response. All other detailed results are shown in Figures S5–S8.

## CONCLUSION

Reasoning GRNs from gene expression data has been a key challenge in systems biology, helping us to identify disease biomarkers and understand gene regulation mechanisms. Current methods for GRN inference from bulk gene expression data usually ignore the regulatory types. However, it is of great significance for cancer-targeted therapy to reconstruct FGRNs with both direction and regulatory types. Thus, this study developed a deep-learning-based model, DeepFGRN, for inferring FGRNs from bulk gene expression profiles. DeepFGRN consists of five submodules: data collection, correlation analysis, node bidirectional representation and prediction and analysis.

With respect to the correlation analysis module, DeepFGRN not only extracts expression embeddings of regulators and target genes via ResNet, but also captures correlation embeddings between regulators and target genes through Cnet. The experimental results verified the importance of correlation embeddings in FGRN inference, which was supported by biological knowledge. In addition, owing to short gene expression data, gradient vanishing is likely to occur, which can be solved using a skip connection. The experimental results validated that the introduction of skip connections can extract better gene expression features.

DeepFGRN establishes the node bidirectional representation module to capture the directed network structure information of FGRNs, which has been verified to be of great importance to the prediction performance. It should be noted that this module designed two generators that could jointly learn the source neighbor embeddings and target neighbor embeddings of each gene node from the same potential continuous distribution. After adversarial learning between the generators and discriminator, their abilities are significantly improved. Consequently, the generators can learn the bidirectional neighbor information for each node, even for those with low in-degrees or out-degrees. Therefore, a node bidirectional representation module can compensate for the shortcomings of sparse and large FGRNs.

Additionally, because there are no benchmark datasets for inferring FGRNs, we collected and preprocess nine benchmark datasets, including simulated datasets (e.g. DREAM5 network1) and experimental datasets (e.g. human breast cancer, liver cancer, lung cancer, COVID-19 and *E. coli* under cold stress, heat stress, lactose and oxidative stress). All datasets were high-dimensional, that is, the number of genes was greater than the number of biological experimental samples. Furthermore, we identified potential biomarkers and drugs for the human breast, liver, lung and COVID-19.

Presently, some methods can only be applied to single static or time-series gene expression data, and the DeepFGRN model based on deep learning methods has no requirements regarding whether the gene expression data are static or temporal. Moreover, DeepFGRN can be implemented in parallel using model parallelism [63]. Specifically, the DeepFGRN model can be divided into multiple sub-models and then trained on different devices (i.e. graphics processing units) in parallel.

In future work, we will consider improving our work from two perspectives to assist in disease treatment. On the one hand, DeepFGRN model is only applicable to bulk gene expression data. Although the model can reconstruct FGRNs from single-cell gene expression data, it may have poor prediction performance because of the high sparsity and high dimensionality of single-cell gene expression data, which the DeepFGRN model fails to solve. Thus, we intend to extend DeepFGRN to GRN inference tasks from single-cell multi-omics sequencing data. On the other hand, we will not only consider the directed network topology information of the GRNs but also consider the functional modules embedded in the GRNs.

**Key Points**

- A deep-learning-based method, DeepFGRN, was proposed for both GRN and FGRN inference, throwing light on the nature of fine gene regulation mechanisms.
- DeepFGRN not only extracts robust features from short gene expression profiles but also considers the correlation between regulators and target genes.
- DeepFGRN embeds a large, sparse and directed FGRN into a low-dimensional, dense and directional space using a GAN with two generators.
- We collect and preprocess nine benchmark datasets, including the DREAM5 challenge, *E. coli* and major human diseases, which are available for FGRN inference by other researchers.

## Supplementary Material

Support_information_for_DeepFGRN_bbae143

## Data Availability

The original data are available from the DREAM5 challenge (https://dreamchallenges.org/dream-5-network-inference-challenge/), RegulonDB (https://regulondb.ccg.unam.mx/index.jsp), Gene Expression Nebulas (GEN) (https://ngdc.cncb.ac.cn/gen/browse/datasets), TRRUST V2 (https://www.grnpedia.org/trrust/) and RegNetwork (https://regnetworkweb.org/home.jsp) databases. DeepFGRN was written in Python and utilized the Keras machine-learning library. PyCharm was used to develop Python. More details, such as the Python libraries and their versions, can be found at https://github.com/PhoebeGaoZhen/DeepFGRN/tree/master, which is available for researchers to reproduce. All processed data, trained models, predictions and README are available at Zenodo DOI: https://zenodo.org/records/10720363.

## Author Biographies

**Zhen Gao** is a PhD candidate at the Key Laboratory of Intelligent Computing and Signal Processing of Ministry of Education, School of Computer Science and Technology, Anhui University, Anhui, China. Her current research interests are in mathematical and computational biology, and systems biology.

**Yansen Su** is a professor at the Key Laboratory of Intelligent Computing and Signal Processing of Ministry of Education, School of Artificial Intelligence, Anhui University, Anhui, China. Her specialized research areas are bioinformatics, systems biology, and artificial intelligence.

**Junfeng Xia** is a professor at Information Materials and Intelligent Sensing Laboratory of Anhui Province, Institute of Physical Science and Information Technology, Anhui University, Anhui, China. His research interests include machine learning and bioinformatics.

**Rui-Fen Cao** is an associate professor at the School of Computer Science and Technology, Anhui University, Anhui, China. Her research interests include artificial intelligence, medical image processing and multimodal data fusion.

**Yun Ding** is a lecturer at the Key Laboratory of Intelligent Computing and Signal Processing of Ministry of Education, School of Artificial Intelligence, Anhui University, Anhui, China. His research interests include computer vision, graph deep learning, single cell omics data analysis.

**Chun-Hou Zheng** is a professor at the Key Laboratory of Intelligent Computing and Signal Processing of Ministry of Education, School of Artificial Intelligence, Anhui University, Anhui, China. His research interests include Pattern Recognition, Intelligent information processing and Bioinformatics.

**Pi-Jing Wei** is a lecturer in the Information Materials and Intelligent Sensing Laboratory of Anhui Province, Institute of Physical Science and Information Technology, Anhui University, Anhui, China. Her main research interests include bioinformatics, synthetic biology, cancer data mining and machine learning.

## References

[1] He W , Tang J, Zou Q, Guo F. MMFGRN: a multi-source multi-model fusion method for gene regulatory network reconstruction. Brief Bioinform 2021;22(6):bbab166. 10.1093/bib/bbab166 33939795

[2] Huynh-Thu VA , Sanguinetti G. Gene regulatory network inference: an introductory survey. In: Sanguinetti G, Huynh-Thu VA (eds). Gene Regulatory Networks. Humana Press, New York, NY: Springer New York LLC, 2019;1883:1–23. 10.1007/978-1-4939-8882-2_1 30547394

[3] Vân Anh Huynh-Thu and Pierre Geurts . dyngenie3: dynamical genie3 for the inference of gene networks from time series expression data. Sci Rep 2018;8(1):1–12. 29467401 10.1038/s41598-018-21715-0 PMC5821733

[4] Huynh-Thu VA , Sanguinetti G. Combining tree-based and dynamical systems for the inference of gene regulatory networks. Bioinformatics 2015;31(10):1614–22. 25573916 10.1093/bioinformatics/btu863 PMC4426834

[5] Zhang Y , Chen Q, Gao D, et al. Grrfnet: Guided regularized random forest-based gene regulatory network inference using data integration. In: 2020 IEEE International Conference on Bioinformatics and Biomedicine (BIBM). Alamitos, CA, USA: IEEE, 2020, p. 132–9.

[6] Zhang Y , Chang X, Liu X. Inference of gene regulatory networks using pseudo-time series data. Bioinformatics 2021;37(16):2423–31. 33576787 10.1093/bioinformatics/btab099

[7] Lei J , Cai Z, He X, et al. An approach of gene regulatory network construction using mixed entropy optimizing context-related likelihood mutual information. Bioinformatics 2022;39:11. 10.1093/bioinformatics/btac717 PMC9805593 36342190

[8] Ma B , Fang M, Jiao X. Inference of gene regulatory networks based on nonlinear ordinary differential equations. Bioinformatics 2020;36(19):4885–93. 31950997 10.1093/bioinformatics/btaa032

[9] Zheng R , Li M, Chen X, et al. An ensemble method to reconstruct gene regulatory networks based on multivariate adaptive regression splines. IEEE ACM Trans Comput Biol Bioinform 2021;18(1):347–54. 30794516 10.1109/TCBB.2019.2900614

[10] Huynh-Thu VA , Irrthum A, Wehenkel L, Geurts P. Inferring regulatory networks from expression data using tree-based methods. PloS One 2010;5(9):e12776. 20927193 10.1371/journal.pone.0012776 PMC2946910

[11] Wang Z , Xu W, San Lucas FA, Liu Y. Incorporating prior knowledge into gene network study. Bioinformatics 2013;29(20):2633–40. 23956306 10.1093/bioinformatics/btt443 PMC3789546

[12] Abbaszadeh O , Azarpeyvand A, Khanteymoori A, Bahari A. Data-driven and knowledge-based algorithms for gene network reconstruction on high-dimensional data. IEEE ACM Trans Comput Biol Bioinform 2022;19(3):1545–57. 33119511 10.1109/TCBB.2020.3034861

[13] Manatakis DV , Raghu VK, Benos PV. Pimgm: incorporating multi-source priors in mixed graphical models for learning disease networks. Bioinformatics 2018;34(17):i848–56. 30423087 10.1093/bioinformatics/bty591 PMC6129280

[14] Gao Z , Tang J, Xia J, et al. Cnngrn: a convolutional neural network-based method for gene regulatory network inference from bulk time-series expression data. IEEE/ACM Trans Comput Biol Bioinform 2023;20(5):2853–61. 37267145 10.1109/TCBB.2023.3282212

[15] Zheng R , Li M, Chen X, et al. BiXGBoost: a scalable, flexible boosting-based method for reconstructing gene regulatory networks. Bioinformatics 2018;35(11):1893–900. 10.1093/bioinformatics/bty908 30395189

[16] Lin Z , Ou-Yang L. Inferring gene regulatory networks from single-cell gene expression data via deep multi-view contrastive learning. Brief Bioinform 2022;24:12. 10.1093/bib/bbac586 36585783

[17] Ma A , Wang X, Wang C, et al. Single-cell biological network inference using a heterogeneous graph transformer. Nat Commun 2023;14:964. 10.1038/s41467-023-36559-0 PMC9944243 36810839

[18] Zhao M , He W, Tang J, et al. A hybrid deep learning framework for gene regulatory network inference from single-cell transcriptomic data. Brief Bioinform 2022;23:01. 10.1093/bib/bbab568 35062026

[19] Hao L , Sun Y, Hao H, et al. Inferring transcription factor regulatory networks from single-cell atac-seq data based on graph neural networks. Nat Mach Intell 2022;4(4):389–400.

[20] Shilu Z , Saptarshi P, Stefan P, et al. Inference of cell type-specific gene regulatory networks on cell lineages from single cell omic datasets. Nat Commun 2023;14:3064. 10.1038/s41467-023-38637-9 PMC10224950 37244909

[21] Radaeva M , Ton A-T, Hsing M, et al. Drugging the ‘undruggable’. Therapeutic targeting of protein–dna interactions with the use of computer-aided drug discovery methods. Drug Discov Today 2021;26(11):2660–79. 34332092 10.1016/j.drudis.2021.07.018

[22] Tian Y , Liu H, Wang M, et al. Role of stat3 and nrf2 in tumors: potential targets for antitumor therapy. Molecules 2022;27(24):8768. 10.3390/molecules27248768 PMC9781355 36557902

[23] Fisher ML , Balinth S, Hwangbo Y, et al. BRD4 regulates transcription factor $\Delta $Np63$\alpha $ to drive a cancer stem cell phenotype in squamous cell carcinomas. Cancer Res 2021;81(24):6246–58. 34697072 10.1158/0008-5472.CAN-21-0707 PMC8692924

[24] Chu P-C , Yu-Chieh W, Chen C-Y, et al. Novel HIF-1$\alpha $ inhibitor CDMP-TQZ for cancer therapy. Future Med Chem 2021 PMID: 33896195; 13(12):1057–72. 33896195 10.4155/fmc-2020-0307

[25] Pei H , Guo W, Peng Y, et al. Targeting key proteins involved in transcriptional regulation for cancer therapy: current strategies and future prospective. Med Res Rev 2022;42(4):1607–60. 35312190 10.1002/med.21886

[26] Xie D , Pei Q, Li J, et al. Emerging role of e2f family in cancer stem cells. Front Oncol 2021;11:723137. 10.3389/fonc.2021.723137 PMC8406691 34476219

[27] Lei X , Zhang M, Guan B, et al. Identification of hub genes associated with prognosis, diagnosis, immune infiltration and therapeutic drug in liver cancer by integrated analysis. Hum Genomics 2021;15(1):1–21. 34187556 10.1186/s40246-021-00341-4 PMC8243535

[28] Yong L , Tang S, Haixin Y, et al. The role of hypoxia-inducible factor-1 alpha in multidrug-resistant breast cancer. Front Oncol 2022;12:964934. 10.3389/fonc.2022.964934 PMC9393754 36003773

[29] López-Menéndez C , Vázquez-Naharro A, Santos V, et al. E2a modulates stemness, metastasis, and therapeutic resistance of breast cancer. Cancer Res 2021;81(17):4529–44. 34145034 10.1158/0008-5472.CAN-20-2685 PMC7611611

[30] Aliya S . Targeting key transcription factors in hepatocellular carcinoma. Crit Rev Oncog 2021;26(1):51–60. 33641284 10.1615/CritRevOncog.2020036027

[31] Zhu S , Li J, Peng H, et al. Adversarial directed graph embedding. In: Proceedings of the Thirty-Fifth AAAI Conference on Artificial Intelligence. Menlo Park, CA: AAAI, 2021;35(5):4741–8.

[32] Xia C , Dong X, Li H, et al. Cancer statistics in China and United States, 2022: profiles, trends, and determinants. Chin Med J (Engl) 2022;135(5):584–90. 35143424 10.1097/CM9.0000000000002108 PMC8920425

[33] Sun Y , Tao Q, Cao Y, et al. Kaempferol has potential anti-coronavirus disease 2019 (covid-19) targets based on bioinformatics analyses and pharmacological effects on endotoxin-induced cytokine storm. Phytother Res 2023;37(6):2290–304. 36726236 10.1002/ptr.7740

[34] Jozefczuk S , Klie S, Catchpole G, et al. Metabolomic and transcriptomic stress response of escherichia coli. Mol Syst Biol 2010;6:364. 20461071 10.1038/msb.2010.18 PMC2890322

[35] Zhang Y , Zou D, Zhu T, et al. Gene expression nebulas (gen): a comprehensive data portal integrating transcriptomic profiles across multiple species at both bulk and single-cell levels. Nucleic Acids Res 2022;50(D1):D1016–24. 34591957 10.1093/nar/gkab878 PMC8728231

[36] Gama-Castro S , Salgado H, Santos-Zavaleta A, et al. Regulondb version 9.0: high-level integration of gene regulation, coexpression, motif clustering and beyond. Nucleic Acids Res 2016;44(D1):D133–43. 26527724 10.1093/nar/gkv1156 PMC4702833

[37] Han H , Cho J-W, Lee S, et al. Trrust v2: an expanded reference database of human and mouse transcriptional regulatory interactions. Nucleic Acids Res 2018;46(D1):D380–6. 29087512 10.1093/nar/gkx1013 PMC5753191

[38] Liu Z-P , Wu C, Miao H, Hulin W. Regnetwork: an integrated database of transcriptional and post-transcriptional regulatory networks in human and mouse. Database 2015;2015:bav095. 26424082 10.1093/database/bav095 PMC4589691

[39] Marbach D , Costello JC, Küffner R, et al. Wisdom of crowds for robust gene network inference. Nat Methods 2012;9(8):796–804. 22796662 10.1038/nmeth.2016 PMC3512113

[40] Schlegl T , Seeböck P, Waldstein SM, et al. f-anogan: fast unsupervised anomaly detection with generative adversarial networks. Medical Image Anal 2019;54:30–44. 10.1016/j.media.2019.01.010 30831356

[41] Wang C , Zhang J, Cheng L, et al. Dpprom: a two-layer predictor for identifying promoters and their types on phage genome using deep learning. IEEE J Biomed Health Inform 2022;26(10):5258–66. 35867364 10.1109/JBHI.2022.3193224

[42] Li W , Zhang W, Zhang J. A novel model integration network inference algorithm with clustering and hub genes finding. Molecular informatics 2020;39(5):e1900075. 31990443 10.1002/minf.201900075

[43] Petralia F , Wang P, Yang J, Zhidong T. Integrative random forest for gene regulatory network inference. Bioinformatics (Oxford, England) 2015;31(12):i197–205. 26072483 10.1093/bioinformatics/btv268 PMC4542785

[44] Mall R , Cerulo L, Garofano L, et al. Rgbm: regularized gradient boosting machines for identification of the transcriptional regulators of discrete glioma subtypes. Nucleic Acids Res 2018;46(7):e39. 29361062 10.1093/nar/gky015 PMC6283452

[45] Sławek J , Ennet TA. Inferring large gene regulatory networks from expression data using gradient boosting. BMC Syst Biol 2013;7:106. 24148309 10.1186/1752-0509-7-106 PMC4015806

[46] Wang J , Ma A, Ma Q, et al. Inductive inference of gene regulatory network using supervised and semi-supervised graph neural networks. Comput Struct Biotechnol J 2020;18:3335–43. 33294129 10.1016/j.csbj.2020.10.022 PMC7677691

[47] Peignier S , Calevro F. Gene self-expressive networks as a generalization-aware tool to model gene regulatory networks. Biomolecules 2023;13(3):526. 36979461 10.3390/biom13030526 PMC10046116

[48] Alawad DM , Ataur Katebi M, Kabir WU, Hoque MT. Agrn: accurate gene regulatory network inference using ensemble machine learning methods. Bioinf Adv 2023;3(1):vbad032. 10.1093/bioadv/vbad032 PMC10082608 37038446

[49] Shannon P , Markiel A, Ozier O, et al. Cytoscape: a software environment for integrated models of biomolecular interaction networks. Genome Res 2003;13, 11:2498–504. 14597658 10.1101/gr.1239303 PMC403769

[50] Chin C-H , Chen S-H. Hsin-hung Wu, Chin-wen Ho, Ming-tat ko, and Chung-yen Lin. Cytohubba: identifying hub objects and sub-networks from complex interactome. BMC Syst Biol 2014;8(Suppl 4):S11. 25521941 10.1186/1752-0509-8-S4-S11 PMC4290687

[51] Chen EY , Tan CM, Kou Y, et al. Enrichr: interactive and collaborative html5 gene list enrichment analysis tool. BMC Bioinformatics 2013;14:128. 23586463 10.1186/1471-2105-14-128 PMC3637064

[52] Kuleshov MV , Jones MR, Rouillard AD, et al. Enrichr: a comprehensive gene set enrichment analysis web server 2016 update. Nucleic Acids Res 2016;44(W1):W90–7. 27141961 10.1093/nar/gkw377 PMC4987924

[53] Xie Z , Bailey A, Kuleshov MV, et al. Gene set knowledge discovery with enrichr. Curr Protocol 2021;1(3):e90. 10.1002/cpz1.90 PMC8152575 33780170

[54] Yoo M , Shin J, Kim J, et al. Dsigdb: drug signatures database for gene set analysis. Bioinformatics 2015;31(18):3069–71. 25990557 10.1093/bioinformatics/btv313 PMC4668778

[55] Qian X , Zhang J, Liu J. Tumor-secreted pge2 inhibits ccl5 production in activated macrophages through camp/pka signaling pathway. J Biol Chem 2011;286(3):2111–20. 21097507 10.1074/jbc.M110.154971 PMC3023508

[56] Banik U , Parasuraman S, Adhikary AK, Othman NH. Curcumin: the spicy modulator of breast carcinogenesis. J Exp Clin Cancer Res 2017;36:98. 28724427 10.1186/s13046-017-0566-5 PMC5517797

[57] Lau GTY , Huang H, Lin S-m, Leung LK. Butein downregulates phorbol 12-myristate 13-acetate-induced cox-2 transcriptional activity in cancerous and non-cancerous breast cells. Eur J Pharmacol 2010;648(1–3):24–30. 20826149 10.1016/j.ejphar.2010.08.015

[58] Jimenez T , Barrios A, Tucker A, et al. Dusp9-mediated reduction of perk1/2 supports cancer stem cell-like traits and promotes triple negative breast cancer. Am J Cancer Res 2020;10(10):3487–506. 33163285 PMC7642669

[59] Rezano A , Ridhayanti F, Rangkuti AR, Gunawan T. Gatot Nyarumenteng a Winarno, and Indra Wijaya. Cytotoxicity of simvastatin in human breast cancer mcf-7 and mda-mb-231 cell lines. Asian Pac J Cancer Prev 2021;22(S1):33–42. 33576210 10.31557/APJCP.2021.22.S1.33

[60] Yin L , He Z, Yi B, et al. Simvastatin suppresses human breast cancer cell invasion by decreasing the expression of pituitary tumor-transforming gene 1. Front Pharmacol 2020;11:574068. 33250768 10.3389/fphar.2020.574068 PMC7672329

[61] Rumgay H , Murphy N, Ferrari P, Soerjomataram I. Alcohol and cancer: epidemiology and biological mechanisms. Nutrients 2021;13(9):3173. 34579050 10.3390/nu13093173 PMC8470184

[62] Bader GD , Hogue CWV. An automated method for finding molecular complexes in large protein interaction networks. BMC Bioinformatics 2003;4:2. 12525261 10.1186/1471-2105-4-2 PMC149346

[63] Nagrecha K . Systems for parallel and distributed large-model deep learning training. CoRR 2023;abs/2301.02691.

