# Supplementary material for: DeepFGRN: inference of gene regulatory network with regulation type based on directed graph embedding
Source: Brief Bioinform. 2024 Apr 4;25(3):bbae143. doi: 10.1093/bib/bbae143 (PMC10998536; doi:10.1093/bib/bbae143)
Supplement: Support_information_for_DeepFGRN_bbae143 [file support_information_for_deepfgrn_bbae143.docx]

**Support information for “DeepFGRN: a deep learning-based model for reconstructing fine gene regulatory network”**

Zhen Gao^1^, Yansen Su^2^, Junfeng Xia^3^, Rui-Fen Cao^1^, Yun Ding^2^, Chun-Hou Zheng^2*^ and Pi-Jing Wei^3*^

1 The Key Laboratory of Intelligent Computing and Signal Processing of Ministry of Education, School of Computer Science and Technology, Anhui University, Hefei 230601, China.

2 The Key Laboratory of Intelligent Computing and Signal Processing of Ministry of Education, School of Artificial Intelligence, Anhui University, Hefei 230601, China.

3 Information Materials and Intelligent Sensing Laboratory of Anhui Province, Institute of Physical Science and Information Technology, Anhui University, 230601, China.

Email:[zhengch99@126.com](mailto:zhengch99@126.com), [weipj@ahu.edu.cn](mailto:weipj@ahu.edu.cn).

**Catalogue**

**Fig. S1** E. coli data collection process.

**Fig. S2** Different architectures of correlation analysis modules.

**Fig.** **S3** The prediction performance of DeepPGRN model using different feature dimensions.

**Fig. S4** Analysis of node bidirectional representation module.

**Fig. S5** Results of enrichment analysis of human breast cancer.

**Fig**. **S6** Results of enrichment analysis of human liver cancer.

**Fig**. **S7** Results of enrichment analysis of human lung cancer.

**Fig. S8** Results of enrichment analysis of human COVID-19.

**Table. S1** Results of different correlation analysis module.

**Table. S2** Experiment settings of node bidirectional representation module.

**Table. S3** Average AUC of five-fold cross-validation for different architectures

**Table.** **S4** Optimal feature dimension for each dataset.

**Table.** **S5** Details of each network in DREAM5 challenge.

**Table. S6** The average AUC value of existing methods and DeepFGRN model under ten times FCV.

**Table. S7** The experimental results reconstructed via DeepFGRN of regular GRN on DREAM5.

**Table. S8** Top ten candidate drugs for human breast cancer, liver cancer, lung cancer and COVID-19 obtained through DSigDB.

**Table.** **S9** Top three functional modules of human breast cancer, liver cancer, lung cancer and COVID-19 obtained through MCODE.

**Text. S1** Performance evaluation metrics

**Text. S2.** Potential biomarkers and drugs analysis

**Supplementary Figures:**


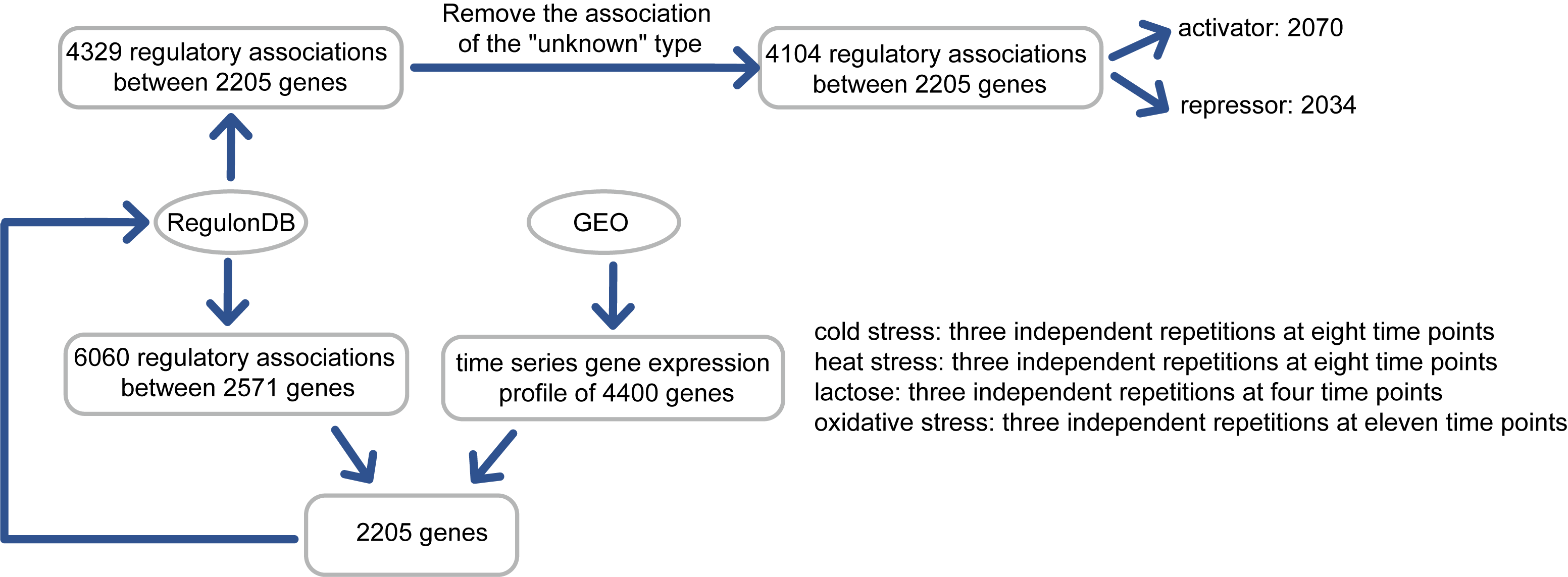


**Fig. S1. E. coli data collection process.** First, download regulatory associations with regulatory types. The experimental dataset is downloaded from the RegulonDB database, which contains a total of 6060 regulatory associations between 2571 genes (including 229 regulator and 2520 target genes) of E. coli, with association types including activation (+), repression (-), and unknown. Second, download time series gene expression data. The experimental dataset is downloaded from the GEO database (accession: GSE20305), including the time series gene expression profile of 4400 genes, all of which are subjected to four different environmental disturbances, including cold stress, heat stress, glucose-lactose diauxic shift, and oxidative stress, and each experimental condition is independently repeated three times at 8, 8, 4, and 11-time points, respectively. Third, Match. Matching 2571 genes in the RegulonDB database (step a.) with 4400 genes in the GEO database (step b) yields 2205 gene names. Finally, retrieval and preprocessing. A total of 4329 known regulatory associations between 2205 genes are retrieved from the RegulonDB database. Then, because unknown types have very few associations, these edges are removed. In the end, we obtain 4,104 regulator-target gene associations between 2,205 genes, of which 2,070 associations are activators and 2,034 associations are repressors.


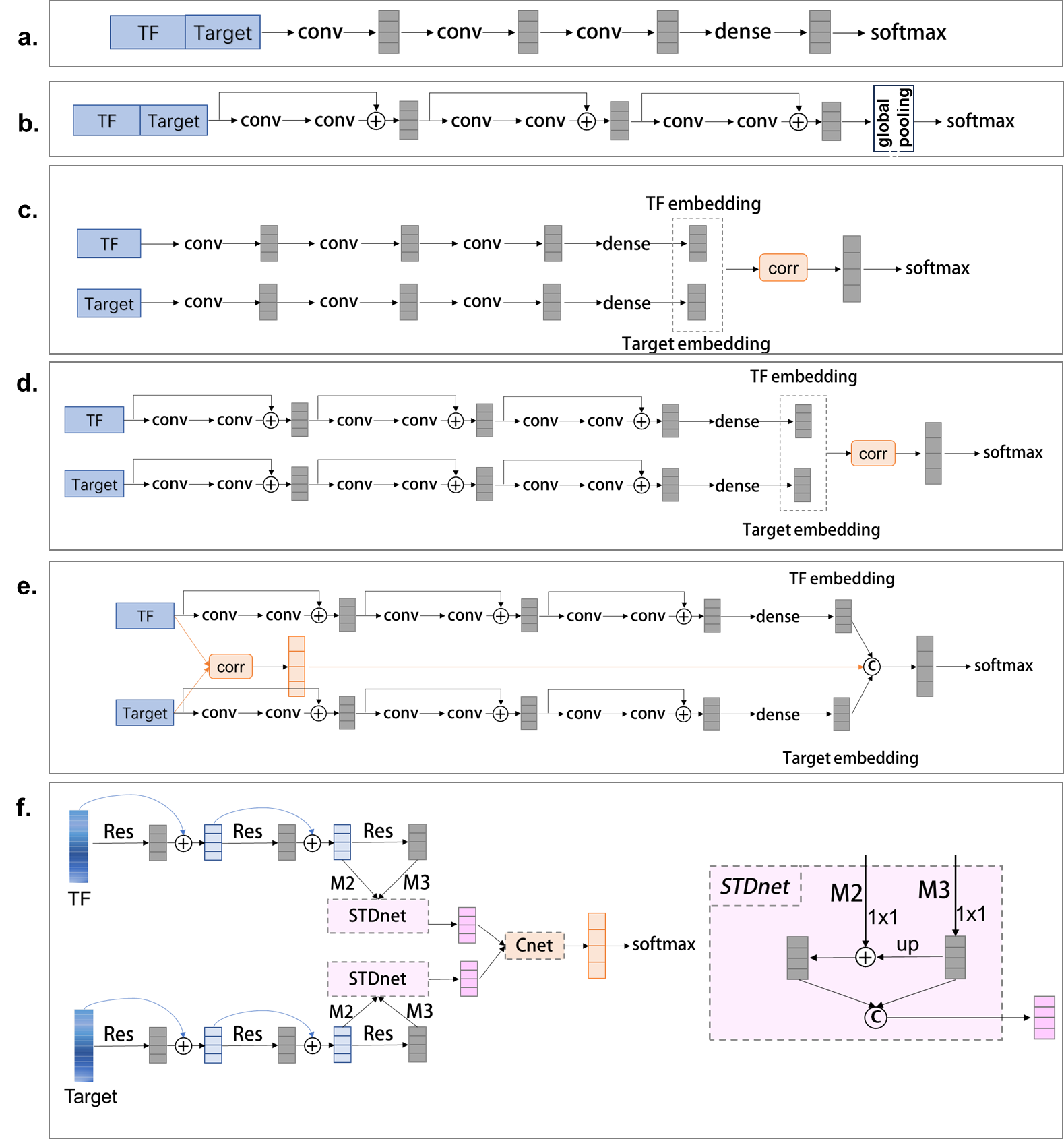


**Fig. S2. Different architectures of correlation analysis modules.** a. expCNN: only used one-dimensional CNN to extract features of gene expression data. b. expResNet: only used one-dimensional ResNet to extract features of gene expression data. c. Cnet: only utilized correlation embedding between regulator and target genes. d. expResNet+Cnet: jointly used one-dimensional ResNet to extract features of gene expression data and correlation embedding between regulator and target genes. e. The correlation embedding learned via gene expression data of regulators and target genes, then combine the correlation embedding, TF embedding and target gene embedding extracted by one-dimensional ResNet. The results are shown in S1 Table.


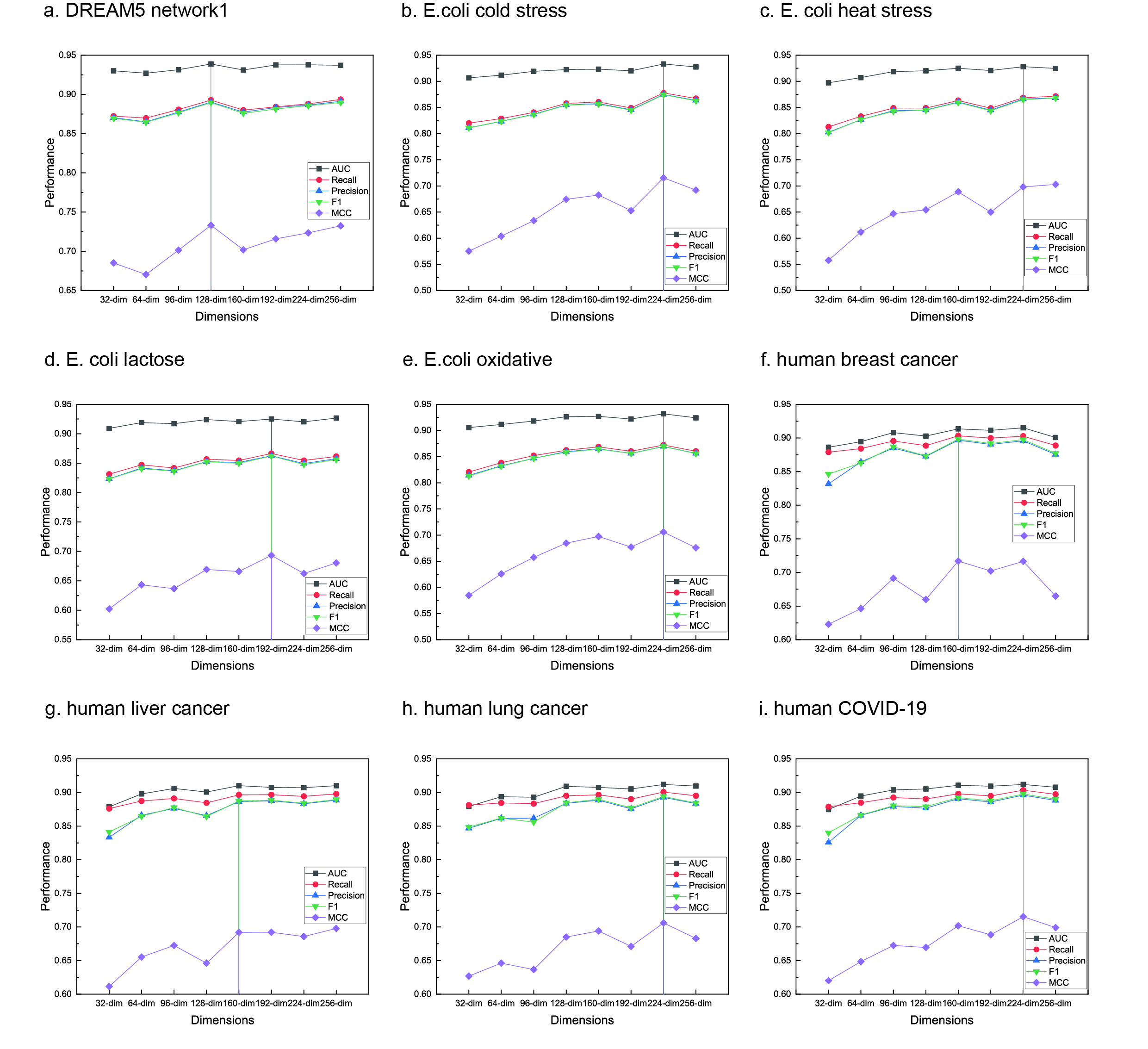


**Fig.** **S3. The prediction performance of DeepPGRN model using different feature dimensions.** The correlation embeddings between regulators and target genes, bidirectional representation of regulators and target genes were acquired via DeepPGRN, and the dimensions of the above features were the same. To get the optimal feature dimension, we implemented five times FCV on all datasets under different experiment settings of different feature dimensions. It can be found that the change of AUC value in different dimensions is not obvious, while the change of MCC value is more sensitive in the change of feature dimension. As a result, we chose the dimensions that could make each index the highest or converge, as shown in S4 Table.


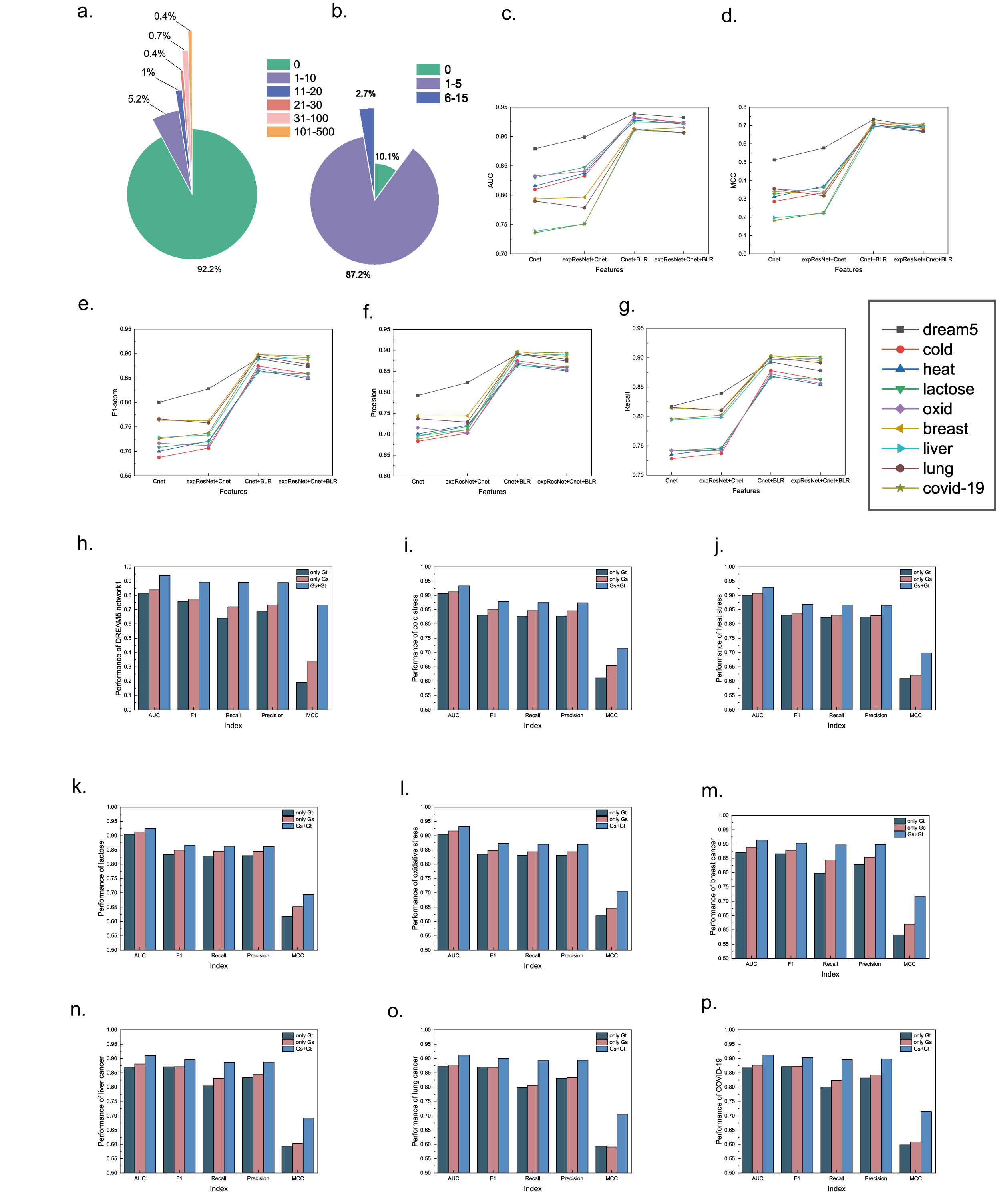


**Fig. S4. Analysis of node bidirectional representation module.** a-b. The out-degree and in-degree of prior FGRN of E.coli. c-g. Average results of ten times FCV for FGRNs inference using four features. h-p. Average results of ten times FCV for FGRNs inference using features of neighbors in different directions.


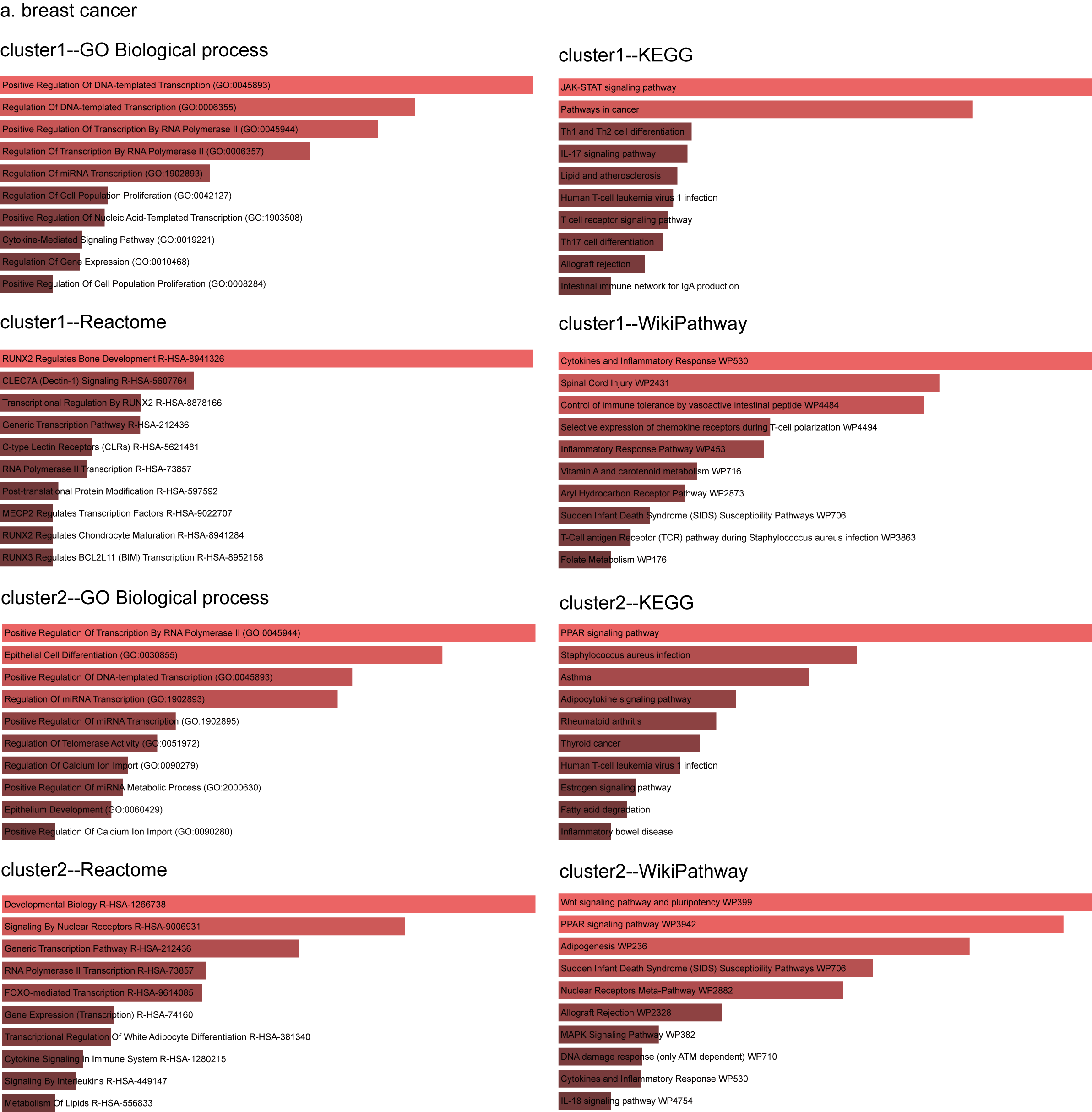


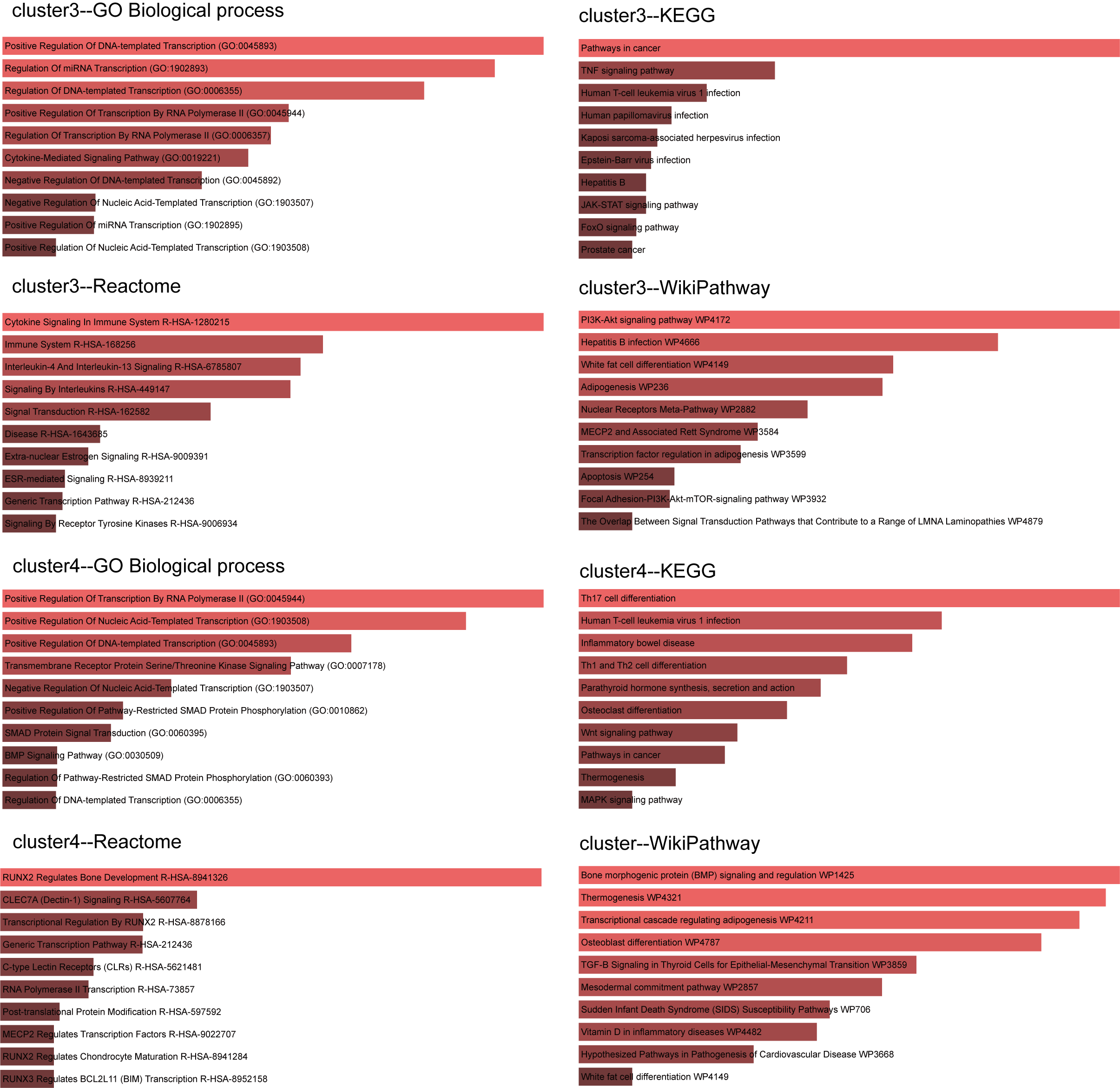


**Fig. S5. Results of enrichment analysis of human breast cancer.**


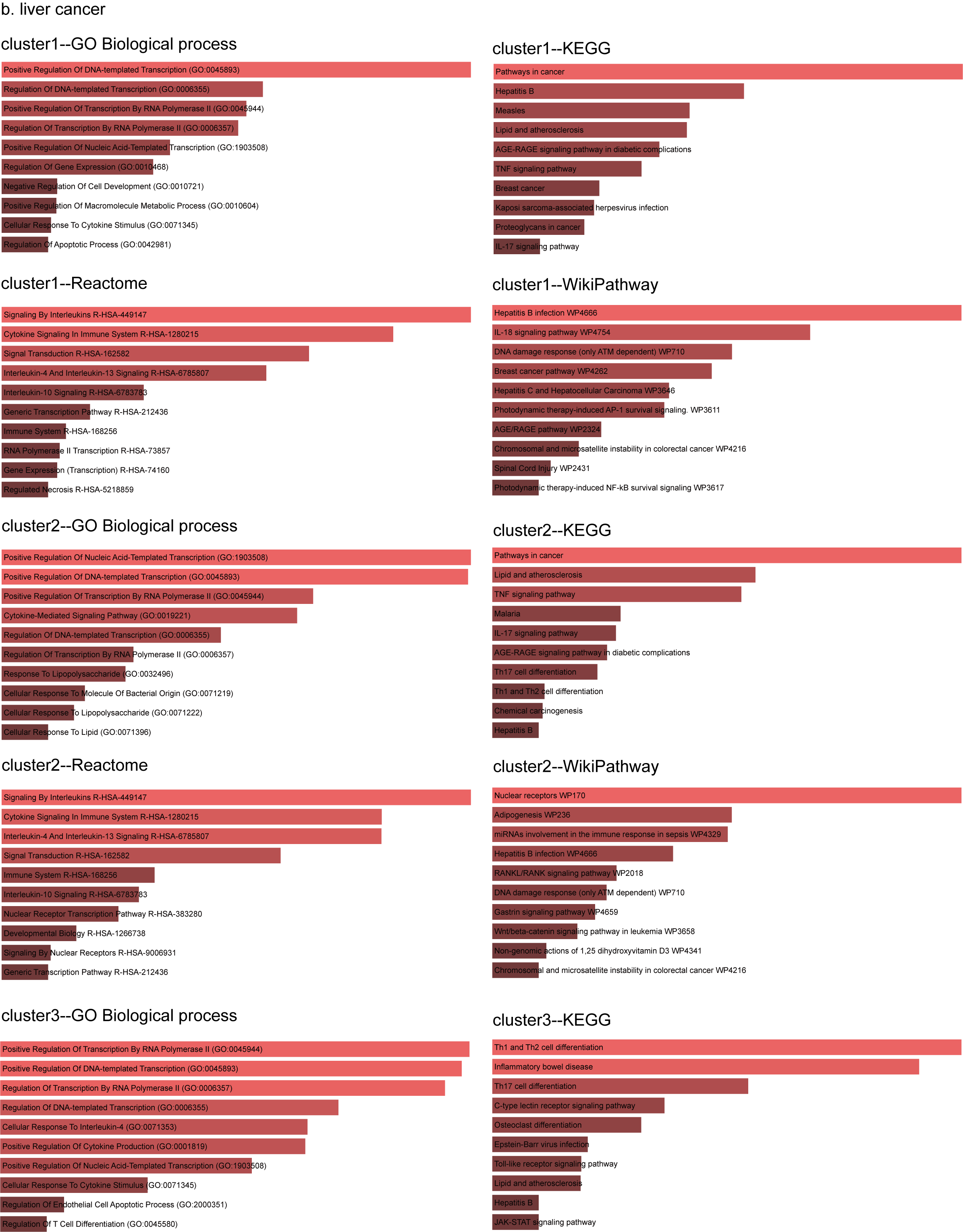


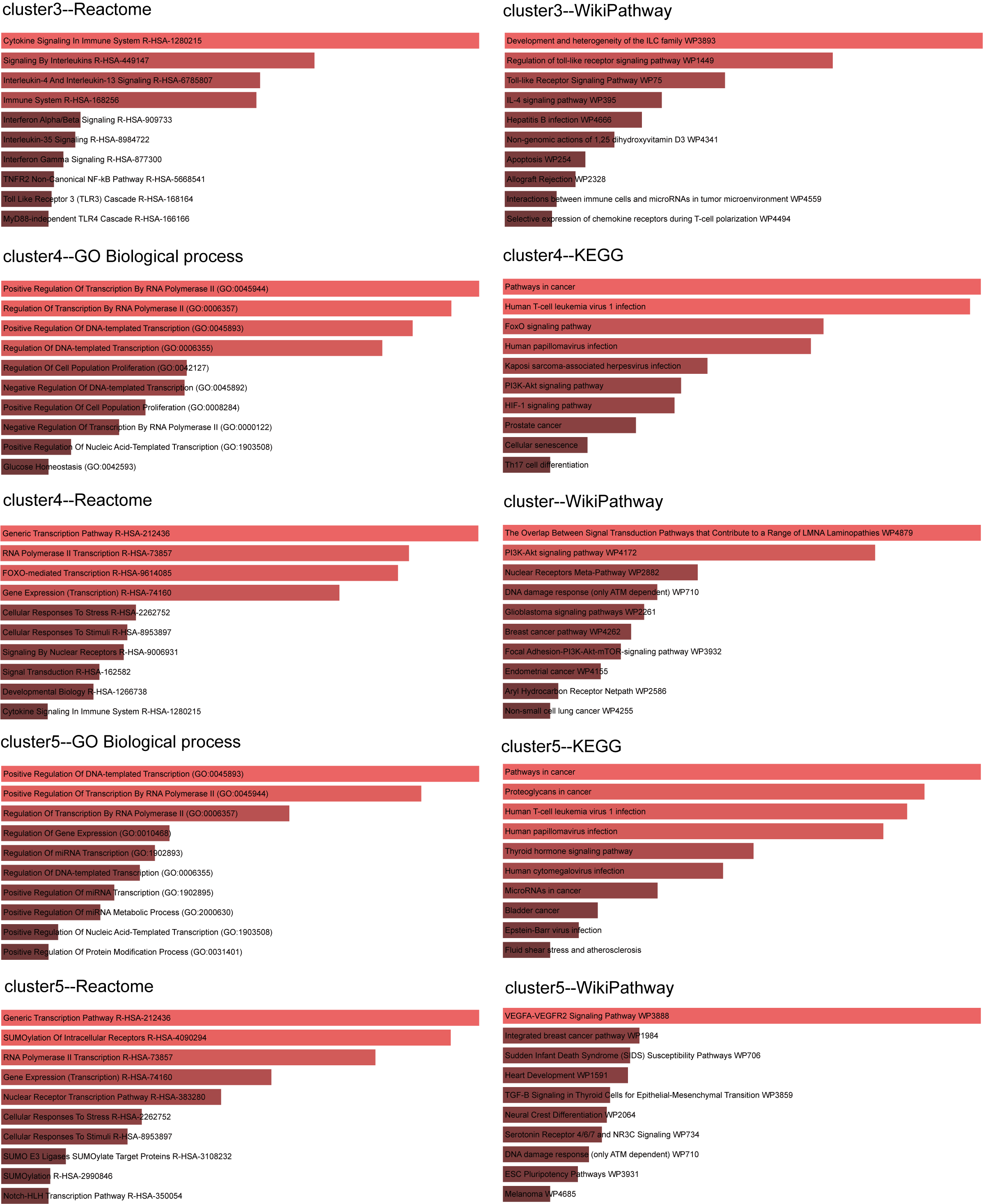


**Fig**. **S6. Results of enrichment analysis of human liver cancer.**


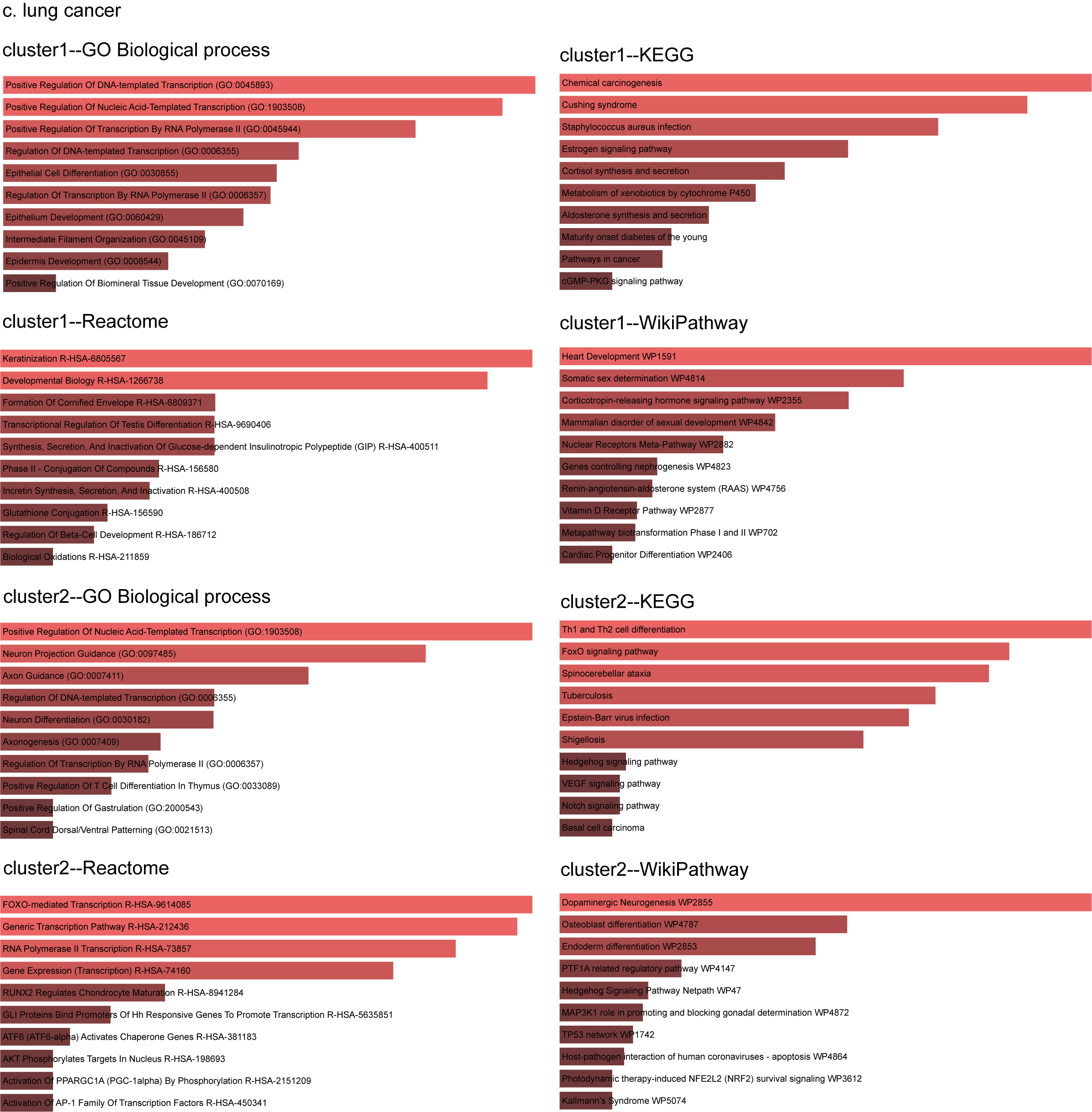


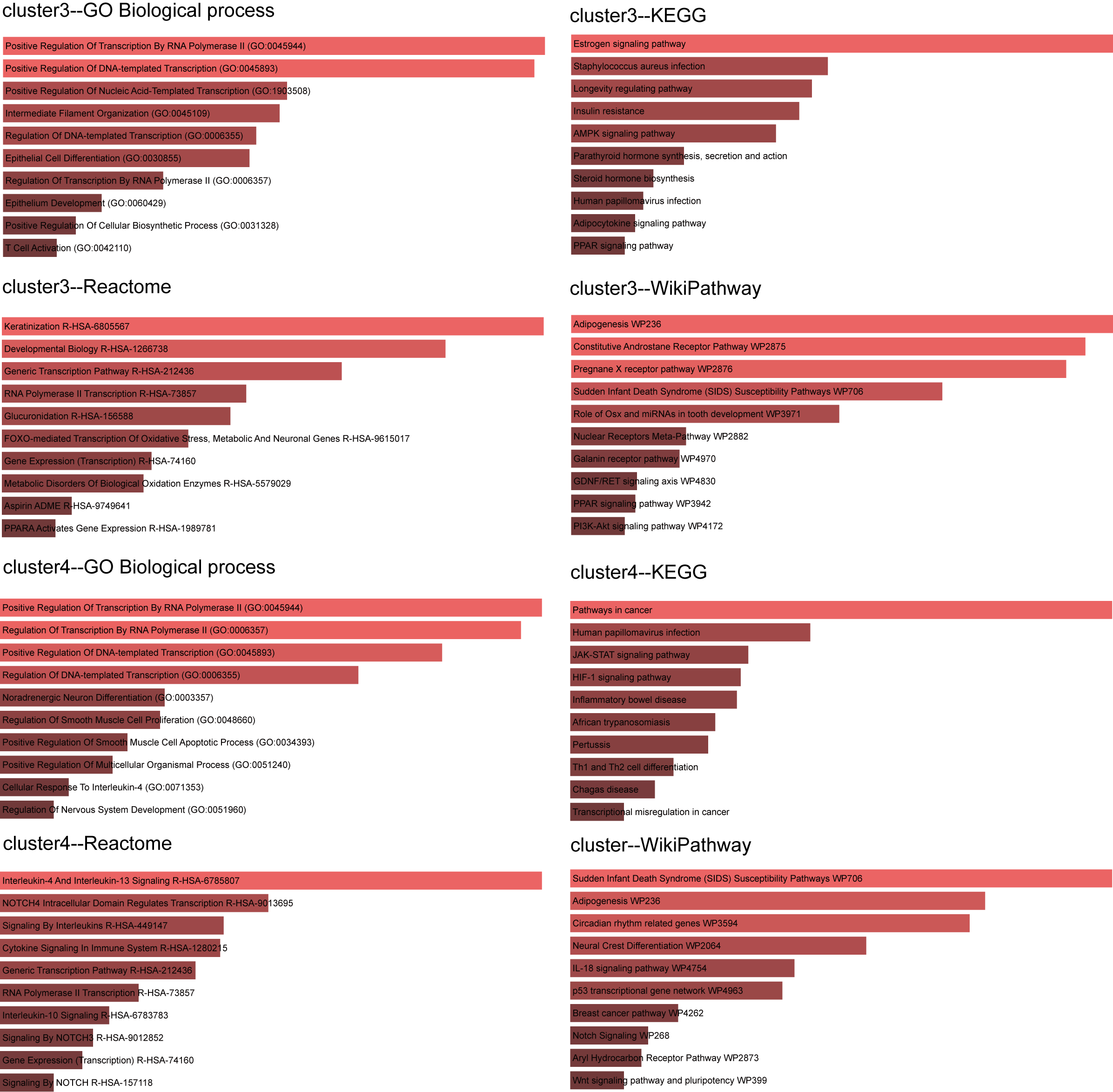


**Fig**. **S7. Results of enrichment analysis of human lung cancer.**


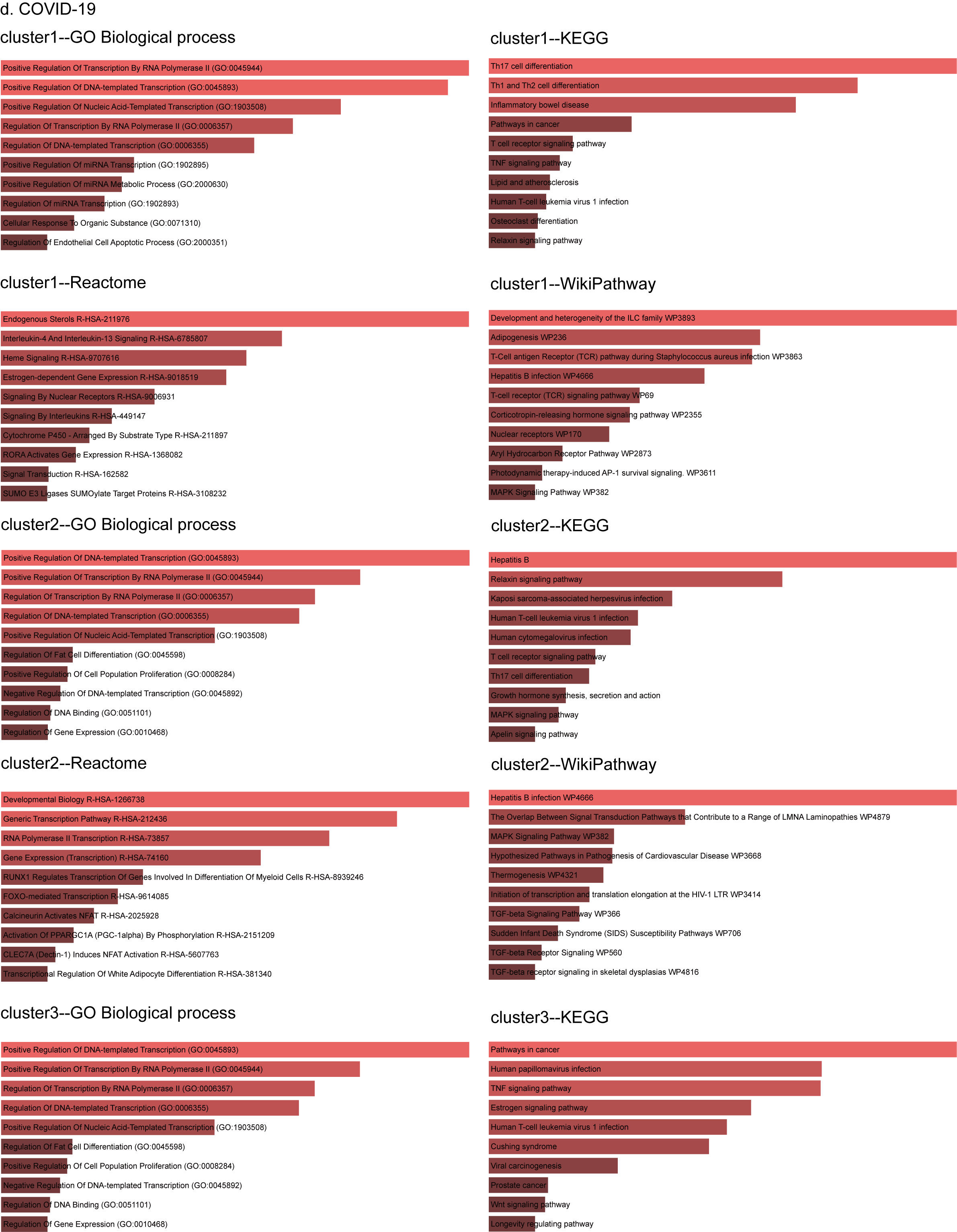


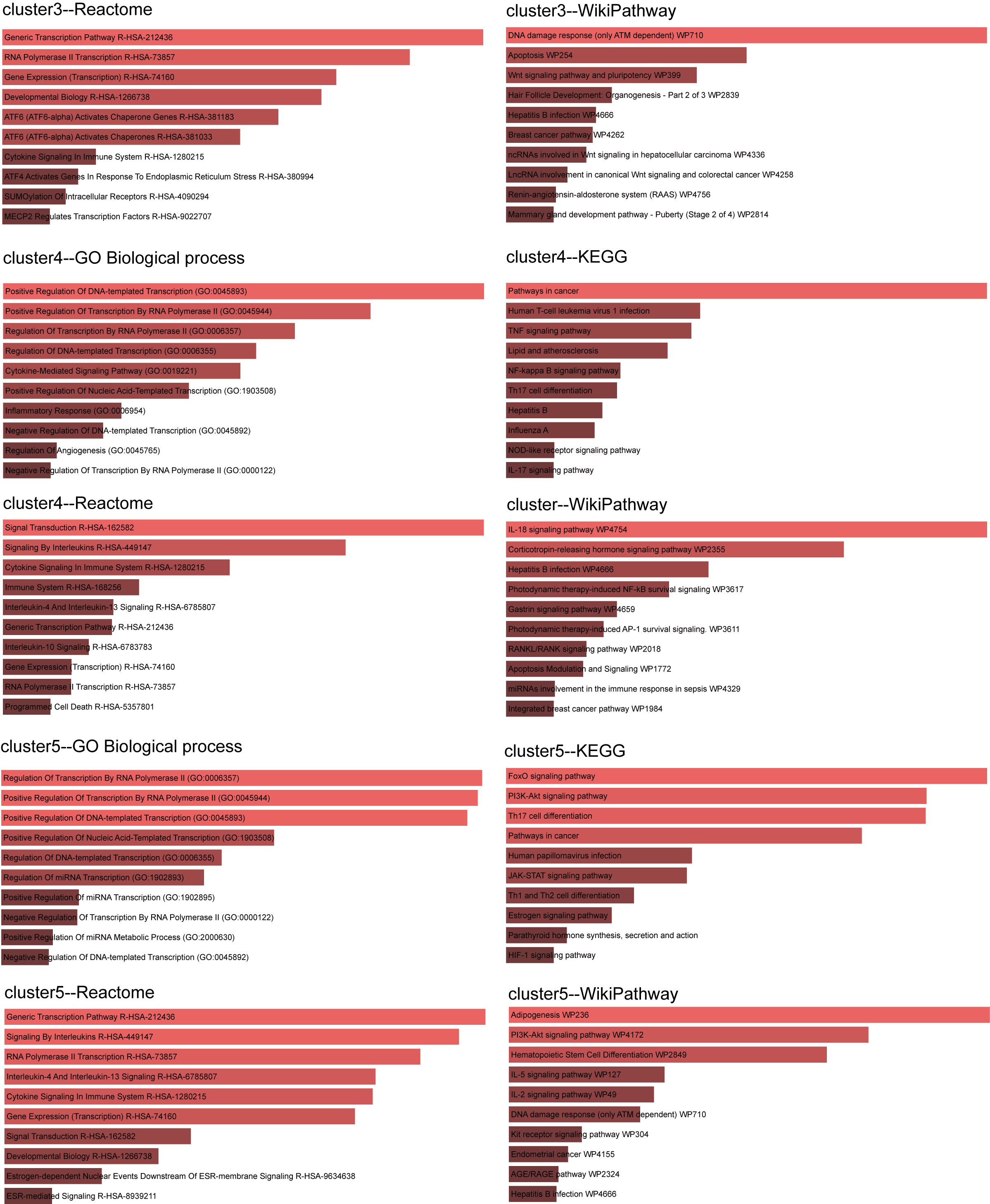


**Fig. S8. Results of enrichment analysis of human COVID-19.**

**Supplementary Tables:**

**Table. S1. Results of different correlation analysis module.** a. expCNN: only used one-dimensional CNN to extract features of gene expression data. b. expResNet: only used one-dimensional ResNet to extract features of gene expression data. c. Cnet: only utilized correlation embedding between regulator and target genes. d. expResNet+Cnet: jointly used one-dimensional ResNet to extract features of gene expression data and correlation embedding between regulator and target genes. e. The correlation embedding learned via gene expression data of regulators and target genes, then combine the correlation embedding, TF embedding and target gene embedding extracted by one-dimensional ResNet.

|  | a | b | c | d | e |
| --- | --- | --- | --- | --- | --- |
| cold | 0.6911±0.0195 | 0.7702±0.0088 | 0.7802±0.0142 | **0.8159±0.0092** | 0.8079±0.0089 |
| heat | 0.6830±0.0129 | 0.7666±0.0111 | 0.7748±0.0103 | **0.8111±0.0147** | 0.8061±0.0109 |
| lactose | 0.7290±0.0196 | 0.8009±0.0118 | 0.7956±0.0132 | **0.8361±0.0138** | 0.8300±0.005 |
| oxidative | 0.7597±0.0143 | 0.8067±0.0151 | 0.8105±0.0083 | **0.8303±0.0122** | 0.8241±0.0124 |

**Table. S2. Experiment settings of node bidirectional representation module.**

| Parameters | value |
| --- | --- |
| The training batch size of the generator | 4 |
| The training batch size of the discriminator | 4 |
| Adversarial iterations | 4 |
| The number of discriminator iterations | 6 |
| The number of generator iterations | 3 |
| Learning rate of discriminator | 1e-4 |
| Learning rate of generator | 1e-4 |

**Table. S3. Average AUC of five-fold cross-validation for different architectures.**

| ID | G | D | Loss | E. coli | DREAM5 | Human |
| --- | --- | --- | --- | --- | --- | --- |
| EC1 | MLP | MLP | JS | 0.7824±0.0137 | 0.7551±0.0053 | 0.7611±0.0075 |
| EC2 | DeConv | MLP | JS | 0.8386±0.0085 | 0.7789±0.0114 | 0.7820±0.0070 |
| EC3 | DeConv | Conv | JS | 0.8433±0.0080 | 0.7897±0.0133 | 0.7688±0.0078 |
| EC4 | MLP | MLP | W | 0.8211±0.0066 | 0.7642±0.0075 | 0.7820±0.0041 |
| **EC5** | **DeConv** | **Conv** | **W** | **0.8491±0.0049** | **0.8048±0.0101** | **0.8388±0.0060** |
| EC6 | DeConv | Conv | W+SN | 0.8244±0.0045 | 0.8021±0.0070 | 0.8212±0.0067 |
| EC7 | MLP | MLP | W+SN | 0.8396±0.0050 | 0.7639±0.0111 | 0.7835±0.0103 |

Note: G and D are the architectures of generators and discriminator, respectively. DeConv denotes the multi-layer perceptron, Conv denotes the convolution layer, JS and W are loss functions based on JS divergence and Wasserstein distance, respectively. SN is spectral normalization.

To get the effective bidirectional representation of nodes, namely directed network structure feature of a PGRN, we designed seven experiments EC1-EC7 to choose the optimal network architecture, as shown in above table. The architecture of source generator was same as target generator, which can be set up as multi-layer perceptron (MLP) or deconvolution layer, and the architecture of the discriminator can be set up as MLP or convolution layer. Besides, the loss function can choose JS divergence [1], Wasserstein distance [2] and spectral normalization [1]. In addition, only the network topological information of PGRNs was used in experiments EC1-EC7, and no gene expression data was used. Experiments EC1-EC7 were carried out on E.coli, DREAM5 network1 and human disease datasets, and the average experimental results of 10 times FCV are shown in above table. It can be found that the use of deconvolution layer or convolutional layer is better than that of MLP layer in generating or discriminating results. The loss function based on Wasserstein distance is more stable than the loss function based on JS divergence, and the performance of the model is slightly degraded after the introduction of spectral normalization. Therefore, the directed graph embedding of PGRNs based on EC5 architecture can better retain the directed topology information of PGRNs and complete the dimensionality reduction (the embedding dimension is set to 64 in this section).

**Table.** **S4. Optimal feature dimension for each dataset.**

| Datasets | Optimal feature dimension |
| --- | --- |
| DREAM5 network1 | 128-dim |
| E. coli cold stress | 224-dim |
| E. coli heat stress | 224-dim |
| E. coli lactose | 192-dim |
| E. coli oxidative stress | 224-dim |
| Breast cancer | 160-dim |
| Liver cancer | 160-dim |
| Lung cancer | 224-dim |
| COVID-19 | 224-dim |

**Table.** **S5. Details of each network in DREAM5 challenge.**

| Network | Organism | numG | dimE | numKA |
| --- | --- | --- | --- | --- |
| Network 1 | in silico | 1643 | 805 | 4012 |
| Network 2 | S.aureus | 2810 | 160 | 518 |
| Network 3 | E. coli | 4511 | 805 | 2066 |
| Network 4 | S. cerevisiae | 5950 | 536 | 3940 |

Note: numG is the number of genes in the corresponding dataset, dimE is the dimension of gene expression profile, numKA is the number of known regulatory associations.

**Table. S6. The average AUC value of existing methods and DeepFGRN model under ten times FCV.**

| **Methods** | Network1-in silico | Network3-E.coli |
| --- | --- | --- |
| MINICHG[3] | 0.827 | 0.614 |
| GENIE3[4] | 0.829 | 0.612 |
| iRafNet[5] | 0.813 | 0.641 |
| RGBM[6] | 0.846 | 0.633 |
| ENNET[7] | 0.857 | 0.632 |
| GRGNN[8] | / | 0.903 |
| GXN•EN[9] | 0.84 | 0.80 |
| GXN•OMP[9] | 0.80 | 0.82 |
| AGRN[10] | 0.8466 | 0.7044 |
| **DeepFGRN** | **0. 9865** | **0. 989** |

**Table. S7. The experimental results reconstructed via DeepFGRN of regular GRN on DREAM5.**

| Indices | Network1 | Network2 | Network3 | Network4 |
| --- | --- | --- | --- | --- |
| AUC | 0.9865 | 0.9952 | 0.9890 | 0.9879 |
| AUPR | 0.9051 | 0.9586 | 0.9015 | 0.8710 |
| Recall | 0.8378 | 0.9348 | 0.9114 | 0.8048 |
| Precision | 0.9925 | 0.9846 | 0.9863 | 0.9827 |
| F1 | 0.8570 | 0.8918 | 0.8022 | 0.8015 |
| MCC | 0.8411 | 0.9083 | 0.8453 | 0.7976 |

**Table. S8. Top ten candidate drugs for human breast cancer, liver cancer, lung cancer and COVID-19 obtained through DSigDB.**

| Diseases | Term | P-value | Evidence |  |
| --- | --- | --- | --- | --- |
| Breast cancer | AH 6809 | 2.23E-12 | [11] |  |
|  | AH 23848 | 2.23E-12 | [11] |  |
|  | curcumin | 9.62E-12 | [12] |  |
|  | Phorbol 12-myristate 13-acetate | 5.17E-10 | [13] |  |
|  | PD 98059 | 9.59E-10 | [14] |  |
|  | simvastatin | 2.34E-09 | [15,16] |  |
|  | acetaldehyde | 2.54E-09 | [17] |  |
|  | DEOXYNIVALENOL | 2.64E-09 | Unconfirmed |  |
|  | Pregna-4,17(20)-diene-3,16-dione | 2.85E-09 | Unconfirmed |  |
|  | N-Acetyl-L-cysteine | 2.90E-09 | Unconfirmed |  |
| Liver cancer | DEOXYNIVALENOL | 1.38E-11 | [18] | |
|  | CHEMBL460515 | 3.15E-11 | Unconfirmed | |
|  | Pterostilbene | 4.68E-11 | [19,20] | |
|  | Indoprofen | 3.97E-10 | Unconfirmed | |
|  | Curcumin | 9.62E-10 | [21] | |
|  | Resveratrol | 1.23E-09 | [22] | |
|  | Zinc sulfate | 1.66E-09 | Unconfirmed | |
|  | Linalool | 1.84E-09 | [23] | |
|  | Pregna-4,17(20)-diene-3,16-dione | 2.85E-09 | Unconfirmed | |
|  | N-Acetyl-L-cysteine | 2.90E-09 | [24] | |
| Lung cancer | AH 6809 | 9.82E-22 | Unconfirmed | |
|  | AH 23848 | 9.82E-22 | Unconfirmed | |
|  | PD 98059 | 9.79E-17 | Unconfirmed | |
|  | curcumin | 1.51E-16 | [25] | |
|  | N-Acetyl-L-cysteine | 5.25E-16 | [26] | |
|  | Pyrrolidine dithiocarbamate | 5.93E-16 | [27] | |
|  | MG-132 | 1.18E-15 | [28] | |
|  | Capsaicin | 4.88E-15 | [29] | |
|  | acetaldehyde | 4.65E-14 | [30] | |
|  | oxygen | 5.31E-14 | [31] | |
| COVID-19 | curcumin | 9.62E-10 | [32] | |
|  | 1,9-yrazoloanthrone | 1.40E-08 | Unconfirmed | |
|  | 170449-18-0 | 2.84E-08 | Unconfirmed | |
|  | bay 11-7082 | 5.68E-08 | Unconfirmed | |
|  | chitosamine | 6.56E-08 | Unconfirmed | |
|  | bezafibrate | 8.62E-08 | [33,34] | |
|  | Tamibarotene | 8.86E-08 | [35] | |
|  | carbon monoxide | 1.81E-07 | [36] | |
|  | Retinoic acid | 1.90E-07 | [37] | |
|  | Hydroxytyrosol | 2.06E-07 | [38,39] | |

**Table.** **S9. Top three functional modules of human breast cancer, liver cancer, lung cancer and COVID-19 obtained through MCODE.**

| Disease | Cluster | Score | Nodes | Edges | Node IDs |  |
| --- | --- | --- | --- | --- | --- | --- |
| Breast cancer | Cluster1 | 10.615 | 27 | 139 | SIRT1, SOX2, FOSL1, CXCL1, HSPA4, TEAD2, SPI1, SLC5A5, CREBBP, CYP11B1, SOCS2, IL5, SOX9, SCARB1, HES1, NFATC3, CD36, STAT2, SP3, HES4, IL4, PGR, NFYC, EGFR, IL2, XPC, RXRB |  |
|  | Cluster2 | 6.885 | 62 | 221 | ENO2, TNFRSF10B, ENG, PCK1, LGALS3, LY96, MEF2B, ICAM1, JUN, CXCL12, CXCR3, NCOA2, NOTCH2, KRT36, TP53, TNF, IL13, PPARD, SCD5, CCNG2, ABCG1, CPT1A, KRT9, HOXA10, DCT, UCP1, CPT1C, HOXB13, NEUROG3, ACAT2, UGT2B7, NKD1, HLA-DMB, KLF4, PPARGC1A, CISH, PLA2G4C, GABARAPL1, ODC1, PLA2G4E, RXRG, SLC22A2, HLA-DRB1, ACADM, IL2RA, MEN1, ASS1, IL15, CLDN2, CREB3L3, HSD17B2, NFYA, FXR2, PPP2R2B, CTNNB1, PDGFRB, PIK3C3, KRT24, MYC, KRT26, KRT27, RELB |  |
|  | Cluster3 | 5.862 | 88 | 262 | BCL6, MYCN, CEBPD, ENO4, MUC2, POU5F1, PIM1, ILK, TNFSF10, PLIN4, TEAD3, TG, PTEN, FOXA3, ESR1, SAMD4A, HDAC1, IRF1, AXIN2, CDKN2B, IL4R, TCF7L2, ZBTB16, FCER2, CXCL3, FCER1A, BTRC, POMC, IFNA21, ARC, TWIST1, GSTA1, E2F1, IFNA6, MMP9, GLI2, FBXO25, EPO, FOS, CEBPA, SRF, PTGS2, ESR2, CSF3R, HLA-DPB1, EZH2, MAPK10, CDKN1B, NR3C1, CCL2, CREB1, MAPK7, CYP19A1, ANPEP, BMP4, CALCR, ACSL4, FOXO6, AR, EGR1, S1PR1, CTSK, MMP14, ITGB3, IFNA1, STAR, BIRC2, EHHADH, STAT5B, WT1, FADS2, IL22, IRF3, EPAS1, KRT12, ENTPD8, LTA, MYB, MCL1, APLNR, PDGFB, LTBR, SLC2A1, PLAUR, SOD2, DR1, CREB3L1, TRAF5, |  |
| Liver cancer | Cluster1 | 10.627 | 84 | 467 | MYBL2, TNFRSF10B, KISS1, CCL20, MIR21, KLK3, LEF1, TCF7, NCOA1, HES5, NOX5, PIM1, TEAD2, TP53, TDRD1, TEAD3, NPAS2, TG, TSHR, SLC10A1, REST, CYP17A1, ESR1, IL1A, IL1RN, ZNF516, IL6, LDLR, G6PC, VEGFB, JUNB, CXCL2, FCER2, RFX5, TWIST1, FASLG, MAPK3, APC, MMP9, CASP3, RFXANK, YBX1, TERT, FOS, BRCA1, GLI3, IFNK, GSTP1, CDK1, MAPK11, HSD3B2, KLF2, CYP19A1, USF2, NFKB1, PAX6, WNT3A, CD74, AGER, TFAP4, BDKRB2, IL17D, IL12B, MMP14, EIF2AK3, IFNA1, SPP1, CFTR, STARD7, BIRC2, STAT5A, PTHLH, STAT5B, KLF5, FABP1, STAT2, WNT3, ATF2, NFATC4, WWTR1, CCND1, DR1, PTGES, BCL2 | |
|  | Cluster2 | 10.553 | 48 | 257 | PIGR, RXRG, ESR2, GK2, CCL2, ICAM1, JUN, CXCL1, ILK, VEGFD, RARA, IL12A, CALCR, YY1, FOXC2, MAPK8, KRT40, AR, CREB3L2, MC2R, PDX1, STARD4, IL5, BCL2L11, MYD88, PPARG, NFYA, ID4, IL12RB1, WNT1, NR5A2, CD68, VCAM1, CXCL8, IL17F, EPAS1, ENTPD3, IRF4, DSG4, SOCS5, NRF1, CTNNB1, NOTCH1, SLC26A4, CREB3, MYC, FOXO4, CHST4 | |
|  | Cluster3 | 7.1 | 21 | 75 | IL12RB2, TBX21, IRF1, CD40, IDO1, MAPK10, IL4R, NFKB2, STAT1, IRF3, FLI1, HES3, SOCS4, TNF, IL4, XBP1, FOSB, NOTCH3, GATA3, HNF1A, NFATC1, | |
| Lung cancer | Cluster1 | 10.615 | 27 | 138 | GSTA3, MATN1, KRT33B, GATA4, POU1F1, PAX6, FOXC2, GSTT2, KRT9, MC2R, SULT2A1, PLG, STAR, SPRR1B, NEUROG3, WT1, BRS3, NFATC2, DSG4, KRT14, ARNT, WNT6, ERVW-1, SPRR2A, CREB5, RELB, RXRB | |
|  | Cluster2 | 6 | 6 | 16 | NFYA, GLI2, OTX2, RBPJL, FOXO6, MAPK11 | |
|  | Cluster3 | 4.943 | 54 | 131 | KRT31, MIR21, POU5F1, GK2, GJD2, KRT34, DLX5, KRT35, NR5A1, KRT36, PADI1, OPRM1, NR1H3, NPPC, G6PC, SLC51B, UGT1A1, KRT13, UGT1A9, ADIPOQ, IFNA14, PRKAG3, NPY, UGT1A4, PPARGC1A, APOA5, EPO, IFNE, GLI3, MIR10B, KLF14, TBX21, APOBEC3A, CYP11B2, SOST, PTH, CTCFL, WNT3A, CREB3L3, HBE1, NEUROD1, HOXA7, MYH7, IVL, IL17F, KRT12, CTNNB1, NOTCH3, CREB3, POU4F2, IFNB1, PSG5, GATA3, INS | |
| COVID-19 | Cluster1 | 10.833 | 25 | 136 | HNF4A, RXRG, TBX21, JUN, NCOA1, CYP11B2, NEFL, NCOA2, VEGFD, TNF, RELA, CYP11B1, NR4A1, PRKACG, FXR2, MITF, RORA, NFATC2, IL4, NFE2L2, OTX2, RBPJL, ATF6B, GATA3, FOS | |
|  | Cluster2 | 9.286 | 15 | 66 | CEBPB, HSF1, SMAD2, RUNX2, NOS1, SOS2, MAPK14, NFATC3, NR5A1, CREBBP, IRF7, HNF1A, PPARGC1A, PRKACA, NFATC1 | |
|  | Cluster3 | 7.818 | 45 | 177 | IL12RB2, CCND2, BLK, CD40, MAPK10, FOSL1, LEF1, CREB1, TRAF1, ACSL4, KRT40, ASXL1, CTSK, KRT9, PDX1, SELE, FOSL2, ATF4, PPARG, HSD3B1, CIITA, RUNX3, IRF1, G6PC2, PRKACB, THBD, IL12RB1, NR5A2, WNT1, WNT5A, ONECUT1, HES1, TP63, TP73, IRF3, HES3, NFYB, SNAI2, PGR, NFYC, MMP9, ERBB2, HDAC2, RCAN2, CREB5 | |

Supplementary Text

**Text. S1. Performance evaluation metrics**

Five-fold cross-validation (FCV) randomly divide all training samples into five equal sub-datasets. Four of them are regarded as a training set to train the model, and the remaining sub-dataset is used as a test set to verify the performance of the model. In this way, each sub-dataset can be used as a test set in turn for five times, and finally the average result is taken as the result of FCV. To be fairer, FCV are usually performed ten times, that is, repeating the above process ten times, and taking the average results of the ten times as the prediction performance of the model. Then, m samples are sorted in descending order according to the prediction results of the model. Next, the classification threshold is set to the maximum value, that is, all samples are predicted to be negative. In this case, both TPR (true positive rate) and FPR (false positive rate) (equation (1)) are 0, that is, a point with coordinate (0,0) can be obtained. By setting the classification threshold as the predicted score of sample A, a set of values of $FPR_{A}$ and $TPR_{A}$, i.e., points with coordinates of $(FPR_{A},TPR_{A})$, can be obtained. Then, the predicted score of each sample was set as the classification threshold, m points could be obtained, and all points were connected to get the ROC curve. AUC is the area under the ROC curve. The higher the value of AUC, the better the prediction performance of the model.

$$AUC=\frac{2}{n(n-1)}\sum_{i=1}^{n} AUC_{i}$$

where $n$ is the number of classes.

Three-class confusion matrix is as follows:


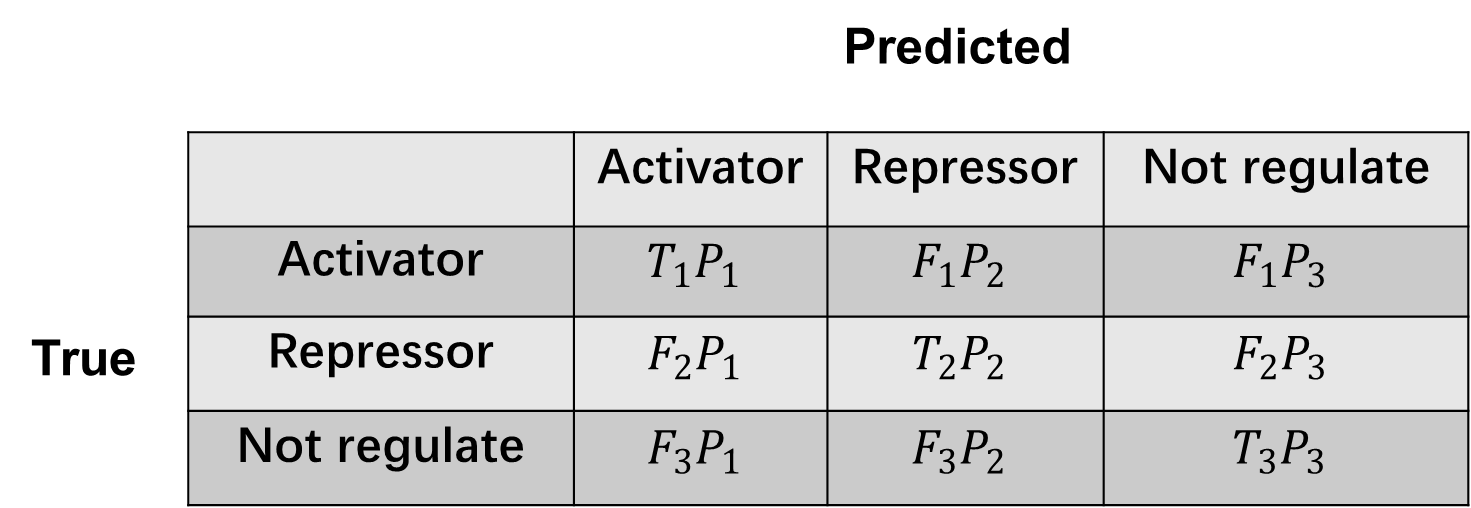

$$TP_{i}=T_{i}P_{i}$$

$$FP_{i}=\sum_{j=1,j\neq i}^{n} F_{j}P_{i}$$

$$FN_{i}=\sum_{j=1,j\neq i}^{n} F_{i}P_{j}$$

where, $TP_{i}$indicates that the true class j is correctly predicted as category i, $F_{j}P_{i}$ indicates that the true class j is incorrectly predicted as category i, $FP_{i}$ indicates the number of samples with incorrect prediction by column, $FN_{i}$ indicates the number of samples with incorrect prediction by row, and n indicates the number of classes.

Precision refers to the proportion of true class *i* in a sample predicted to be class *i*. That is, how many of the samples predicted as class *i* are actually class *i* samples.

$$Precision=\frac{1}{n}\sum_{i=1}^{n} \frac{TP_{i}}{TP_{i}+FP_{i}}$$

Recall refers to the proportion of samples that are actually class i that are predicted to be class i. That is, among the samples that are really class i, how many samples are predicted to be class i.

$$Recall=\frac{1}{n}\sum_{i=1}^{n} \frac{TP_{i}}{TP_{i}+FN_{i}}$$

F1-score refers to the harmonic average of Precision and Recall. Precision and Recall are a pair of contradictory quantities. When Precision is high, Recall will be relatively low, and when Recall is high, Precision will be relatively low. Therefore, in order to better evaluate the performance of the classifier, Generally, F1-Score is used as the evaluation standard to measure the comprehensive performance of the classifier.

$$F1-score=\frac{1}{n}\sum_{i=1}^{n} \frac{2*Precision_{i}*Recall_{i}}{Precision_{i}+Recall_{i}}$$

Matthews Correlation Coefficient (MCC) treats the real and predicted classes as two (binary) variables and calculates their correlation coefficients (similar to calculating the correlation coefficients between any two variables). The higher the correlation between the true value and the predicted value, the better the prediction. The prediction produces a high score only if it achieves good results across all four confusion array classes (TP, TN, FN, and FP).

$$MCC=\frac{TF*TN-FP*FN}{\sqrt{(TP+FP)(TP+FN)(TN+FP)(TN+FN)}}$$

According to the calculation formula, it can be seen that when the classifier is perfect (FP =FN =0), the value of MCC is 1, indicating a complete positive correlation; on the contrary, when the classifier is always wrong (TP = TN =0), the teaching value obtained is -1, representing a perfect negative correlation. Therefore, the value of MCC is always between -1 and 1, and 0 means that the divider is no better than random binary selection. In addition, MCC is completely symmetric, so no one class is more important than the other, and if you switch the positive and negative classes, you still get the same value.

**Text. S2. Potential biomarkers and drugs analysis**

To demonstrate the significance of FGRNs inference in human disease treatment clearly, we identified potential biomarkers and drugs for breast cancer, liver cancer, lung cancer and COVID-19, respectively. Specifically, a pre-trained model is obtained by training the DeepFGRN model using a balanced training set, which includes all prior gene pairs and the same number of unexplored gene pairs. Then, the remaining unexplored gene pairs were fed into the pre-trained DeepFGRN model to obtain the predicted scores. The above gene pairs were sorted in descending order according to their predicted scores. Next, a relatively complete FGRN which close to real was obtained via combining all prior gene pairs together with top *N* unexplored gene pairs. Subsequently, the visualization and bioinformatics analysis were conducted on this relatively complete FGRN.

**References**

1. Miyato T, Kataoka T, Koyama M, Yoshida Y (2018) Spectral Normalization for Generative Adversarial Networks. Arxiv.
2. Arjovsky M, Chintala S, Bottou L (2017) Wasserstein GAN. Arxiv.
3. Li W, Zhang W, Zhang J (2020) A Novel Model Integration Network Inference Algorithm with Clustering and Hub Genes Finding. Molecular Informatics 39.
4. Huynh-Thu VA, Irrthum A, Wehenkel L, Geurts P (2010) Inferring Regulatory Networks from Expression Data Using Tree-Based Methods. Plos One 5.
5. Petralia F, Wang P, Yang J, Tu Z (2015) Integrative random forest for gene regulatory network inference. Bioinformatics 31: 197-205.
6. Mall R, Cerulo L, Garofano L, Frattini V, Kunji K, et al. (2018) RGBM: regularized gradient boosting machines for identification of the transcriptional regulators of discrete glioma subtypes. Nucleic Acids Research 46.
7. Slawek J, Arodz T (2013) ENNET: inferring large gene regulatory networks from expression data using gradient boosting. BMC Systems Biology 7.
8. Wang J, Ma A, Ma Q, Xu D, Joshi T (2020) Inductive inference of gene regulatory network using supervised and semi-supervised graph neural networks. Computational and Structural Biotechnology Journal 18: 3335-3343.
9. Peignier S, Calevro F (2023) Gene Self-Expressive Networks as a Generalization-Aware Tool to Model Gene Regulatory Networks. Biomolecules 13.
10. Alawad DM, Katebi A, Kabir MWU, Hoque MT (2023) AGRN: accurate gene regulatory network inference using ensemble machine learning methods. Bioinformatics advances 3: vbad032-vbad032.
11. Qian X, Zhang J, Liu J (2011) Tumor-secreted PGE(2) Inhibits CCL5 Production in Activated Macrophages through cAMP/PKA Signaling Pathway. Journal of Biological Chemistry 286: 2111-2120.
12. Banik U, Parasuraman S, Adhikary AK, Othman NH (2017) Curcumin: the spicy modulator of breast carcinogenesis. Journal of Experimental & Clinical Cancer Research 36.
13. Lau GTY, Huang H, Lin S-m, Leung LK (2010) Butein downregulates phorbol 12-myristate 13-acetate-induced COX-2 transcriptional activity in cancerous and non-cancerous breast cells. European Journal of Pharmacology 648: 24-30.
14. Jimenez T, Barrios A, Tucker A, Collazo J, Arias N, et al. (2020) DUSP9-mediated reduction of pERK1/2 supports cancer stem cell-like traits and promotes triple negative breast cancer. American Journal of Cancer Research 10: 3487-3506.
15. Rezano A, Ridhayanti F, Rangkuti AR, Gunawan T, Winarno GNA, et al. (2021) Cytotoxicity of Simvastatin in Human Breast Cancer MCF-7 and MDA-MB-231 Cell Lines. Asian Pacific journal of cancer prevention : APJCP 22: 33-42.
16. Yin L, He Z, Yi B, Xue L, Sun J (2020) Simvastatin Suppresses Human Breast Cancer Cell Invasion by Decreasing the Expression of Pituitary Tumor-Transforming Gene 1. Frontiers in Pharmacology 11.
17. Rumgay H, Murphy N, Ferrari P, Soerjomataram I (2021) Alcohol and Cancer: Epidemiology and Biological Mechanisms. Nutrients 13.
18. Claeys L, Romano C, De Ruyck K, Wilson H, Fervers B, et al. (2020) Mycotoxin exposure and human cancer risk: A systematic review of epidemiological studies. Comprehensive Reviews in Food Science and Food Safety 19: 1449-1464.
19. Gomez-Zorita S, Gonzalez-Arceo M, Trepiana J, Aguirre L, Crujeiras AB, et al. (2020) Comparative Effects of Pterostilbene and Its Parent Compound Resveratrol on Oxidative Stress and Inflammation in Steatohepatitis Induced by High-Fat High-Fructose Feeding. Antioxidants 9.
20. Zhan J, Hu T, Shen J, Yang G, Ho C-T, et al. (2021) Pterostilbene is more efficacious than hydroxystilbenes in protecting liver fibrogenesis in a carbon tetracholride-induced rat model. Journal of Functional Foods 84.
21. Li W, Chen Y, He K, Cao T, Song D, et al. (2022) The Apoptosis of Liver Cancer Cells Promoted by Curcumin/TPP-CZL Nanomicelles With Mitochondrial Targeting Function. Frontiers in Bioengineering and Biotechnology 10.
22. Wu S-X, Xiong R-G, Huang S-Y, Zhou D-D, Saimaiti A, et al. (2022) Effects and mechanisms of resveratrol for prevention and management of cancers: An updated review. Critical Reviews in Food Science and Nutrition.
23. An Q, Ren J-N, Li X, Fan G, Qu S-S, et al. (2021) Recent updates on bioactive properties of linalool. Food & Function 12: 10370-10389.
24. Satoh K (2018) Strong carcinogenic stress response induction of preneoplastic cells positive for GST-P in the rat liver: Physiological mechanism for initiation. Life Sciences 200: 42-48.
25. Ashrafizadeh M, Najafi M, Makvandi P, Zarrabi A, Farkhondeh T, et al. (2020) Versatile role of curcumin and its derivatives in lung cancer therapy. Journal of Cellular Physiology 235: 9241-9268.
26. Yao J, Ma C, Gao W, Liang J, Liu C, et al. (2016) Fentanyl induces autophagy via activation of the ROS/MAPK pathway and reduces the sensitivity of cisplatin in lung cancer cells. Oncology Reports 36: 3363-3370.
27. Tahata S, Yuan B, Kikuchi H, Takagi N, Hirano T, et al. (2014) Cytotoxic effects of pyrrolidine dithiocarbamate in small-cell lung cancer cells, alone and in combination with cisplatin. International Journal of Oncology 45: 1749-1759.
28. Li Y, Dong S, Tamaskar A, Wang H, Zhao J, et al. (2020) Proteasome Inhibitors Diminish c-Met Expression and Induce Cell Death in Non-Small Cell Lung Cancer Cells. Oncology Research 28: 497-507.
29. Han T-H, Park MK, Nakamura H, Ban HS (2022) Capsaicin inhibits HIF-1 alpha accumulation through suppression of mitochondrial respiration in lung cancer cells. Biomedicine & Pharmacotherapy 146.
30. Eriksson CJP (2015) Genetic-Epidemiological Evidence for the Role of Acetaldehyde in Cancers Related to Alcohol Drinking. In: Vasiliou V, Zakhari S, Seitz HK, Hoek JB, editors. Biological Basis of Alcohol-Induced Cancer. pp. 41-58.
31. Simeonov KP, Himmelstein DS (2015) Lung cancer incidence decreases with elevation: evidence for oxygen as an inhaled carcinogen. Peerj 2.
32. Sadeghizadeh M, Asadollahi E, Jahangiri B, Yadollahzadeh M, Mohajeri M, et al. (2023) Promising clinical outcomes of nano-curcumin treatment as an adjunct therapy in hospitalized COVID-19 patients: A randomized, double-blinded, placebo-controlled trial. Phytotherapy Research.
33. Rogosnitzky M, Berkowitz E, Jadad AR (2020) Delivering Benefits at Speed Through Real-World Repurposing of Off-Patent Drugs: The COVID-19 Pandemic as a Case in Point. JMIR public health and surveillance 6: e19199-e19199.
34. Rogosnitzky M, Berkowitz E, Jadad AR (2020) No Time to Waste: Real-World Repurposing of Generic Drugs as a Multifaceted Strategy Against COVID-19. JMIRx med 1: e19583-e19583.
35. Mujwar S (2021) Computational repurposing of tamibarotene against triple mutant variant of SARS-CoV-2. Computers in Biology and Medicine 136.
36. Qin W, Chen S, Zhang Y, Dong F, Zhang Z, et al. (2021) Diffusion Capacity Abnormalities for Carbon Monoxide in Patients with COVID-19 At Three-Month Follow-up. European Respiratory Journal 58.
37. Sarohan AR (2020) COVID-19: Endogenous Retinoic Acid Theory and Retinoic Acid Depletion Syndrome. Medical Hypotheses 144.
38. Bonetti G, Medori MC, Fioretti F, Farronato M, Nodari S, et al. (2022) Dietary supplements for the management of COVID-19 symptoms. Journal of preventive medicine and hygiene 63: E221-E227.
39. de la Lastra JMP, Andres CMC, Juan CA, Plou FJ, Perez-Lebena E (2023) Hydroxytyrosol and Arginine as Antioxidant, Anti-Inflammatory and Immunostimulant Dietary Supplements for COVID-19 and Long COVID. Foods 12.
